# Supplementary material for: AI-driven robotic crystal explorer for rapid polymorph identification
Source: Digit Discov. 2026 Jan 2;5(2):734–42. doi: 10.1039/d5dd00203f (PMC13548306; doi:10.1039/d5dd00203f)
Supplement: Supplementary file 1 [file DD-005-D5DD00203F-s001.pdf]

Supplementary Information (SI) for Digital Discovery.
This journal is © The Royal Society of Chemistry 2026

Supplementary Information for:

AI-Driven Robotic Crystal Explorer for Rapid Polymorph Identification

Edward C Lee†, Daniel Salley†, Abhishek Sharma and Leroy Cronin

[1]School of Chemistry, University of Glasgow, University Avenue, Glasgow G12 8QQ, UK.

*Corresponding author Email: Lee.Cronin@glasgow.ac.uk

†These authors contributed equally.

Contents

1. General overview 3
1.1 Platform construction materials/equipment 3
1.1.1 Commercial components 3
1.1.2 3D printing 3
1.1.3 Laser cutting 3
1.2 Platform operation 4
1.3 Experimental apparatus and chemical sources 4
2. Robotic platform 5
2.1 Overview 5
2.2 Platform construction 7
2.2.1 Base frame and working areas 7
2.2.2 Reagent dispensing apparatus 10
2.2.3 Stirring apparatus 11
2.2.4 Imaging apparatus 13
2.2.5 Combined liquid handling and imaging set-up 16
2.2.6 Vial handling 17

3. Crystal Vision and Analysis 21
3.1 Training MARCO dataset clustering 21
3.2 Training ROY dataset-polymorph 24
3.3 Bayesian Algorithm 28
4. References 35

1. General overview

1.1 Platform construction materials/equipment

The platform was constructed using a range of commercially available, 3D printed, and laser cut parts are explained in the details below.

1.1.1 Commercial components

V-slot aluminium profile and associated fixtures were used to construct the frame of the platform. Stepper motors ranging from NEMA 11-17 were employed in all moving component assemblies. Linear rail and guides (NSK Ltd.) were used to direct motion of these assemblies. Raspberry Pi boards, cameras and lens' were used to record imaging data. Arduino MEGA/RAMPs control boards (for stepper motors) were used to control the operations of the physical hardware. Mechanical positioning switches were used to position hardware and a Dell Optiplex PC was used to operate the system. Screws, bearings and other fixtures were bought from UK suppliers such as RS components, Ooznest Ltd, Farnell Ltd and Accu (accu.co.uk).

1.1.2 3D printing

Many custom components were designed, and 3D printed to construct this system. All prints were designed using Onshape CAD software (onshape.com) and printed on a Objet350 Connex3 Stratasys printer. The majority of parts were printed in FullcureRDG720 material with inlays of Agilis rubber material or VeroWhite as for gripping or labelling purposes respectively. It should be noted that the STL files supplied in the repository associated with this work are parts designed in the knowledge that a high calibre 3d printer of this standard was to be used for smooth functioning. This means if other, less capable printers were used to replicate this work, post modification of prints may be needed to accommodate great part allowances. This is noted on the specific parts that are likely to need this attention.

1.1.3 Laser cutting

Laser cutting of structural 2D parts (e.g., vial tray, vial storage outlines) were cut from 4 and 6mm acrylic using an Epilogue Fusion Edge laser cutter (60W laser). Standard operational values for these materials supplied with this machine were sufficient to produce all cut and engraved outlines.

1.2 Platform operation

The working area of the platforms has multiple devices controlled by one of three methods. All stepper motors, stirring mechanisms and linear motion is controlled via Arduino/RAMPs. Each board can control up 5 stepper motors, multiple MOSFET outputs and general position hardware. HD cameras were controlled using Raspberry Pi single board computers. Finally, the Gardener Denver Tricontinent C3000 series syringes pumps used for this work are controlled by a custom designed board via a single USB. Hardware controlled by the Arduino/RAMPs combinations use our in-house software package, Commanduino (https://github.com/croningp/commanduino). The Raspberry Pi on this system is used as a server programmed using sockets, commanded to acquire images at intervals determined by the user. The Tricontinent pumps are controlled using our in-house software, Pycont (https://github.com/croningp/pycont).

1.3 Experimental apparatus and chemical sources

MF-1083 micro vials for electrochemistry from Alvatek were used in all experiments (chosen for the flat, optically clear vial base that was ideal for imaging). HPLC grade solvents were used in all experiments, purchased from Sigma Aldrich§ and used without modification. ROY was purchased from TCI chemicals (Purity > 97%) and used without further purification. Vials were washed in the same HPLC grade acetone used as the primary solvent for stock solution of ROY.

2. Robotic platform

2.1 Overview

The automated platform designed for this work is an extension of the Modular Wheel Platform (MWP) published by our group previously.[1] The MWP is a liquid handling device capable of many simple to moderate complex actions that a human operator would ordinarily perform; dispensing, stirring, sample extraction, pH modification etc. Experiments are performed in parallel, and each experiment can be addressed by any functionality included on the system. This platform incorporated a custom designed vial handling apparatus as part of its workflow, meaning a higher throughput of reactions can be performed as well as introducing the capability of moving reaction vials to other functional operations such as heating mantles. Figure 1 and Figure 2 show the full platform in a CAD rendering and the fully constructed system respectively. The following section details the platforms base construction up to its specific configuration needed to complete this work.

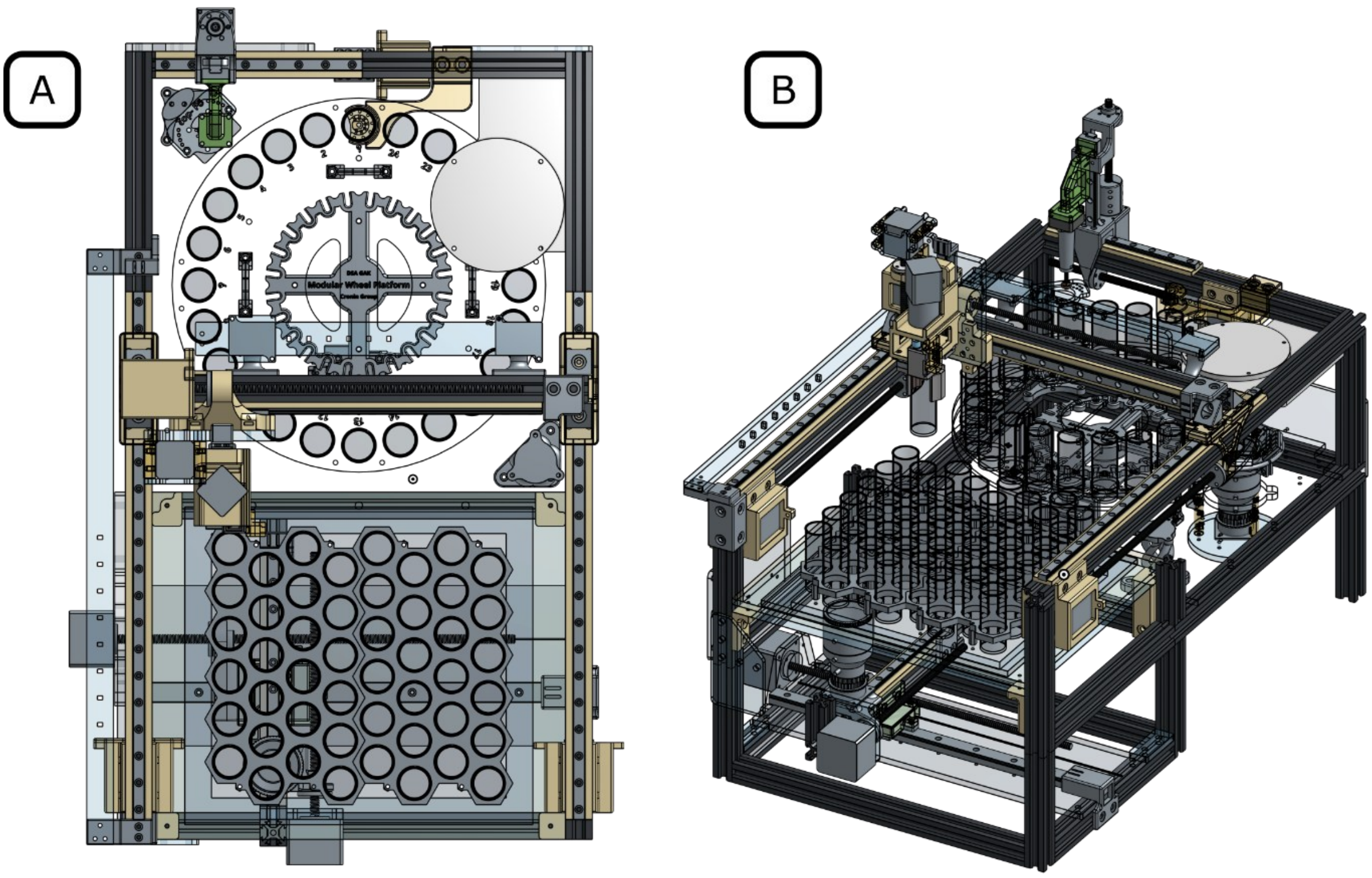

Figure 1. (A) Top CAD view of the automated system. (B) Isometric view of the automated system.

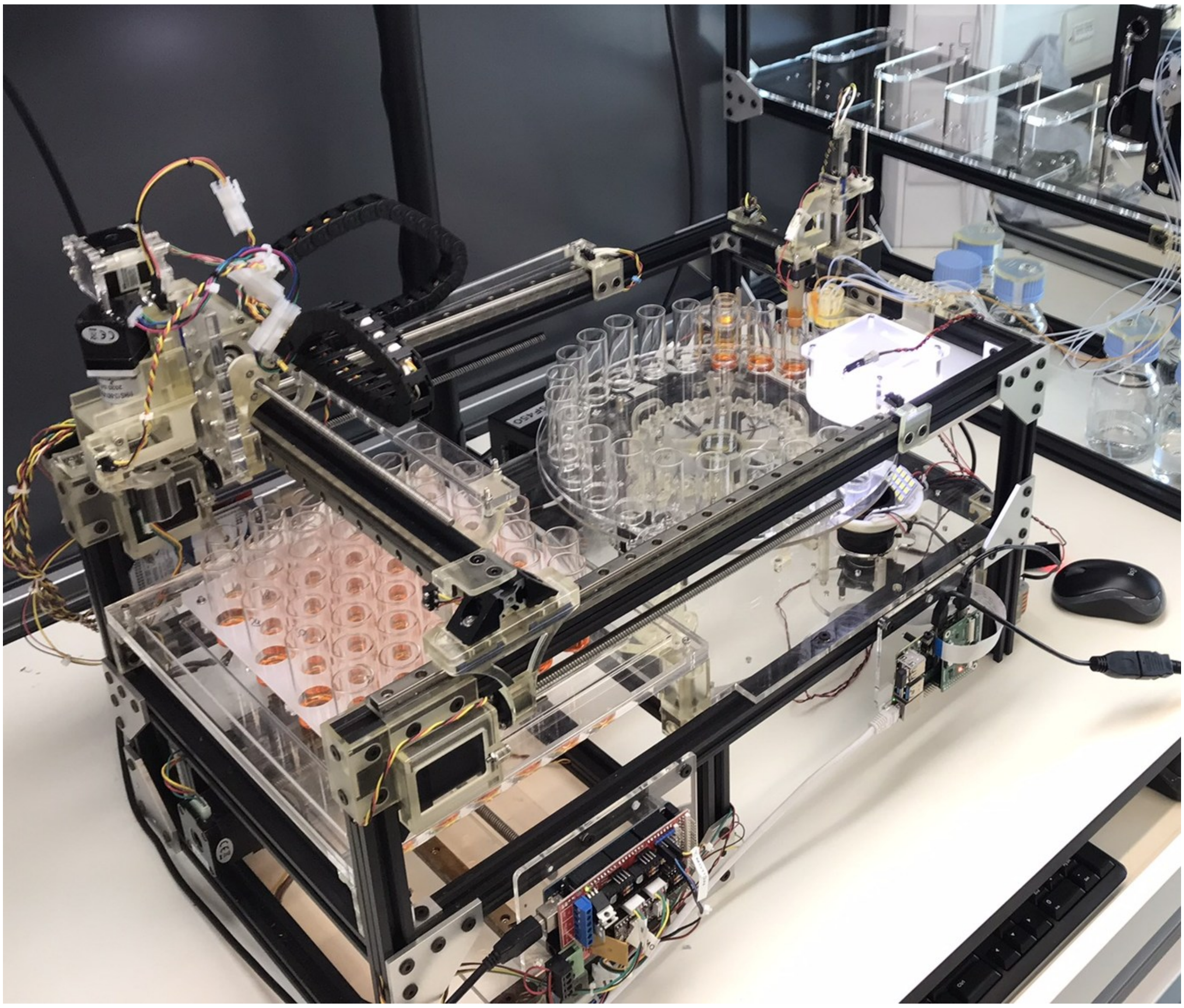

Figure 2. Image of fully constructed automated platform.

2.2 Platform construction

2.2.1 Base frame and working areas

The platform structural frame is constructed from stand 20×20mm v-slot aluminium profile. can be thought of as having two working areas that can be customized as needed:

1. The chemistry handling/imaging area.
2. Vial storage area with moving gripper.

Figure 3 shows these two areas from above. The gripping mechanism links the two areas but the clarity in figures/descriptions, the gripping mechanism is included as part of the storage area.

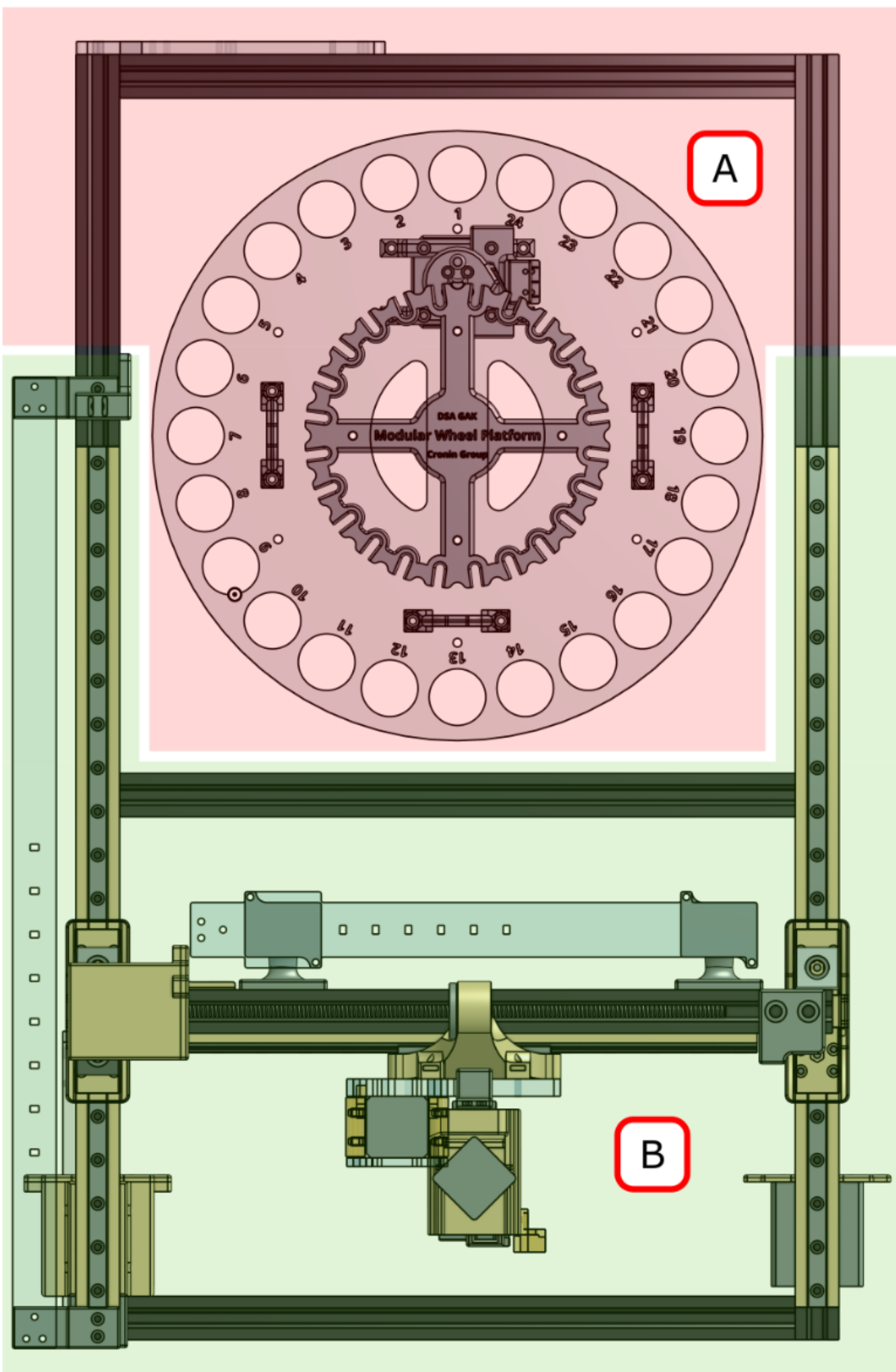

Figure 3. Platform seen from above (CAD). (A) Chemical handling and imaging area. (B) Vial storage area with vial handing gripper.

In the area 1, reagents are dispensed and mixed from above, completing 24 samples per series. Imaging of the samples is also conducted here once crystallisation is complete. Area 2 is used as a storage area for crystallizing samples. As discussed in the main text, 24 samples are performed in triplicate for each generation of this work. Once complete the first series of 24 samples are removed from area 1 to area 2 by a mobile gripper, followed by the second complete series and the finally 24 samples are left on the wheel area during crystallisation.

Figure 4 shows area 1 in greater detail. As stated earlier this platform is an extension of the MWP seen in other published work, this section of the platform is an embedded MWP, driven by a 15° Geneva wheel mechanism. Details included here concern only the addition modules to the system needed for this work. For construction details of the MWP please see previous work.[1]

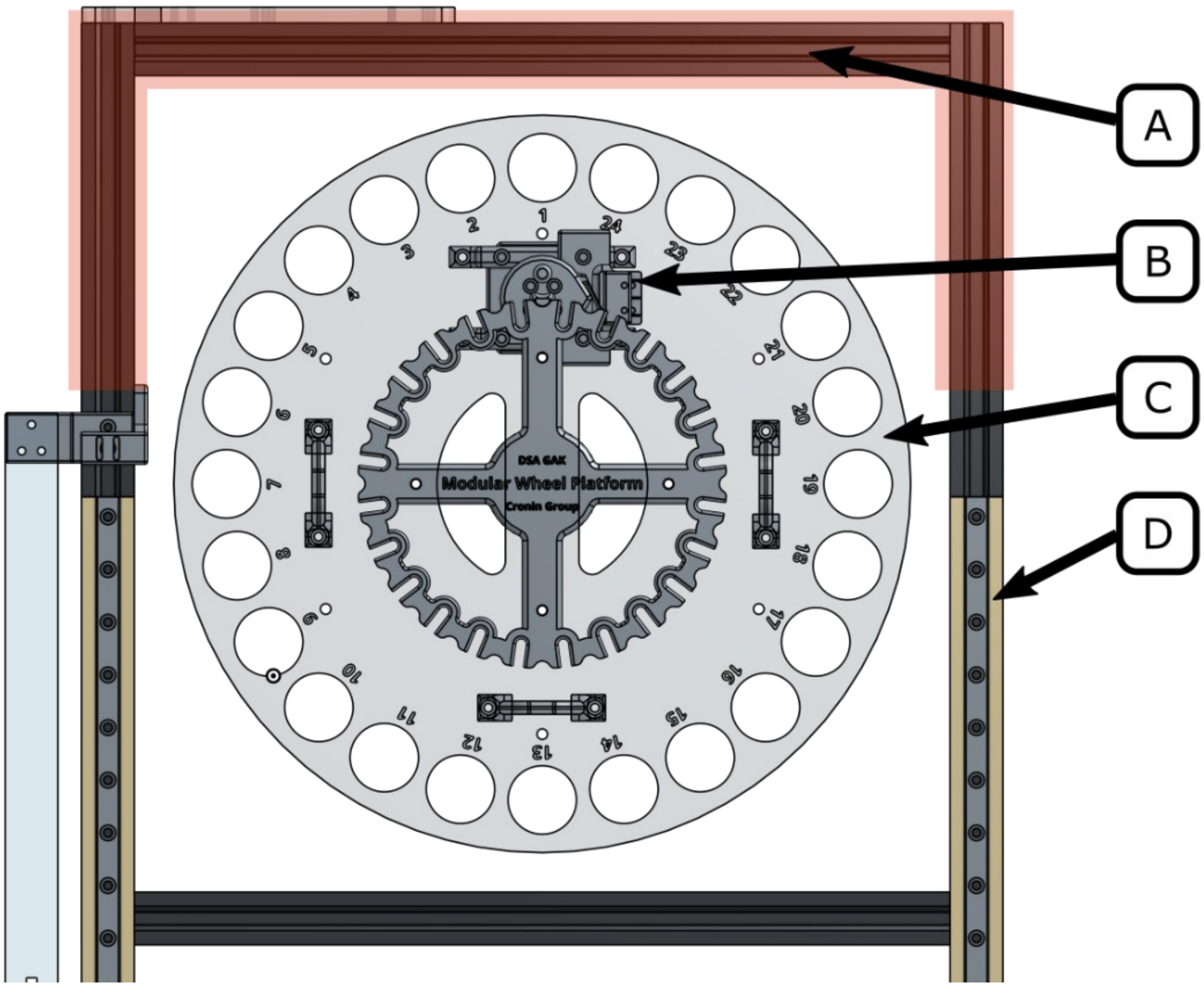

Figure 4. Embedded MWP section of the platform. (A) Customizable area surrounding the wheel. (B) Geneva drive with endstop positioning. (C) Laser cut acrylic vial tray (labelled engraved for imaging). (D) Linear rails extending into storage area.

Figure 5 shows the storage area with the moving customizable stage detail. The gripping mechanism is attached to this stage and can be seen in Figure 6.

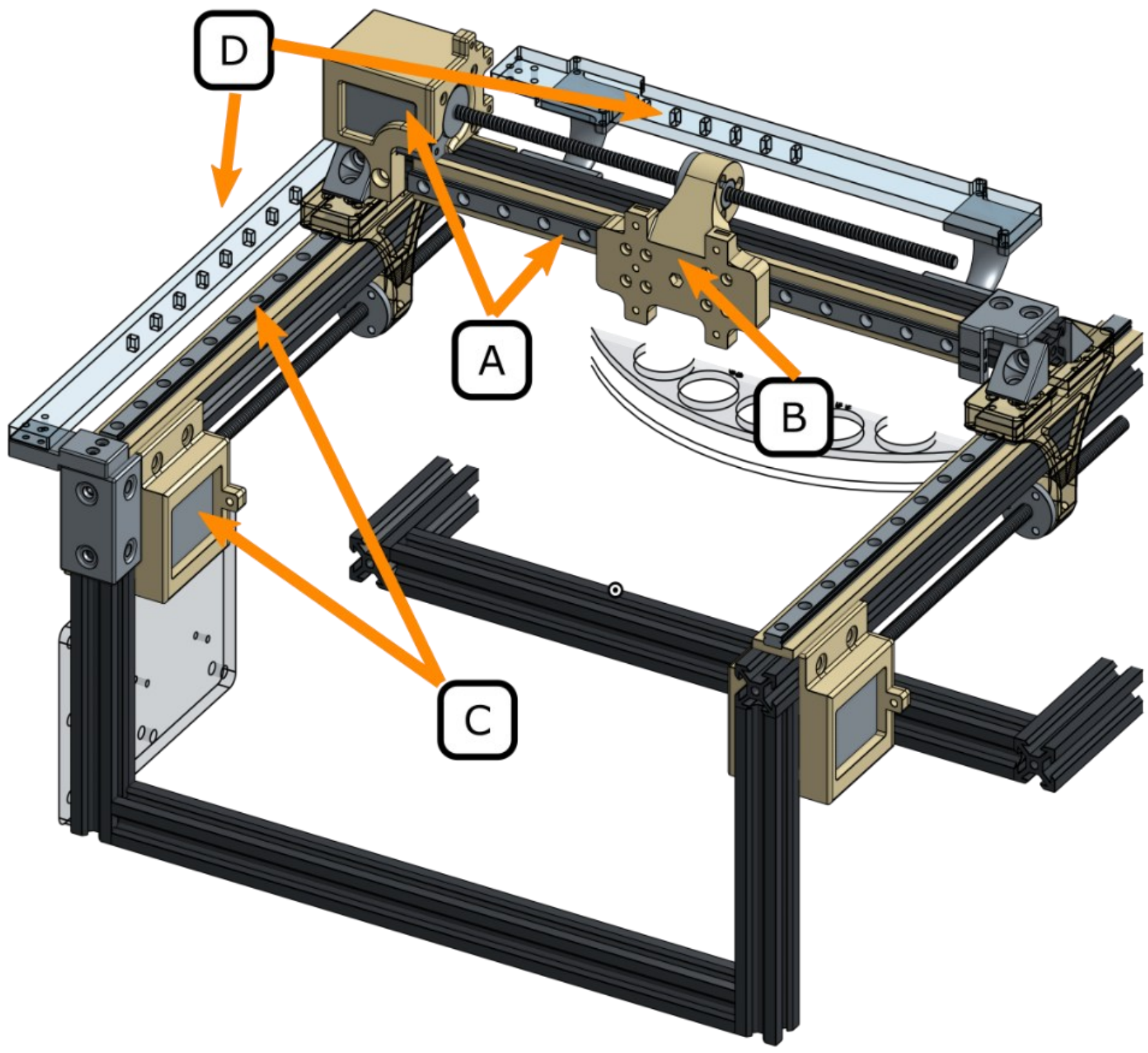

Figure 5. Storage area and mobile axis (area 2). (A) X-axis lead screw + motor with linear rail. (B) X-motion base stage. (C) Y-axis lead screw motor and linear rail. (D) Cable chain mounts.

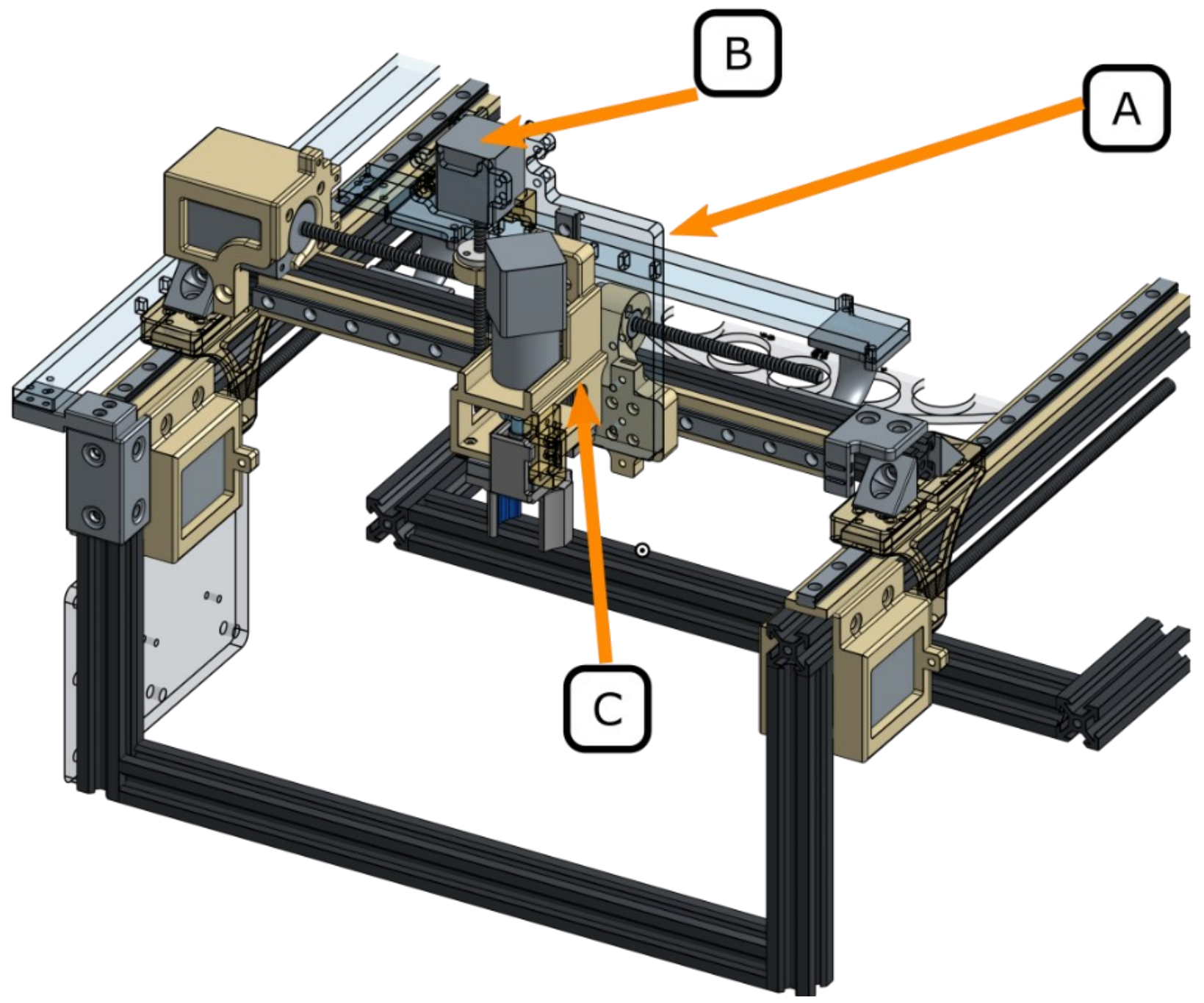

Figure 6. Storage area and gripper. (A) Base plate of gripper mechanism bound directly to the x-motion base stage (B) Nema 11 lead screw motor to control z-motion of gripper. (C) Gripper main body.

2.2.2 Reagent dispensing apparatus

As mentioned earlier, samples are prepared on the embedded MWP of this platform. Reagents are dispensed through a 3D printed module, using Tricontinent syringe pumps, into flat bottom clear vials see Figure 7.

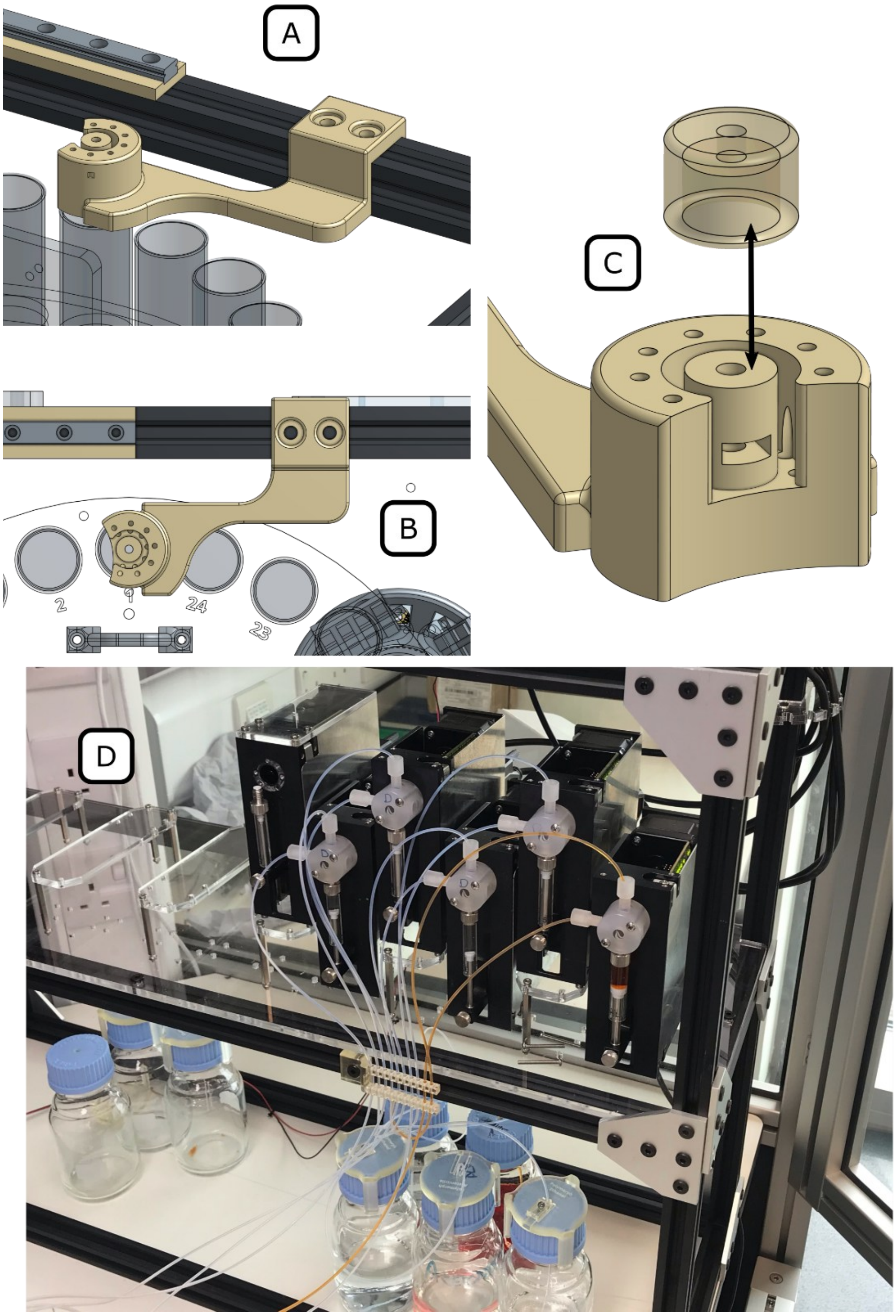

Figure 7. Dispensing apparatus. (A) Printed dispensing unit (isometric projection). (B) Printed dispensing unit top view over Microcell MF-1052 vials. (C) Securing nut for reagent tubing. (D) Tricontinent pumps and stock solutions.

NB Microcell MF-1052 vials were selected for this work due to the near perfect clarity of the vial base. Ordinary chemistry vials have significant rippling in the base glass piece which would distort the crystal images significantly.

2.2.3 Stirring apparatus

Once reagents are dispensed it is critical to sufficiently mix the solutions, however conventional stirring using a magnetic stir bar would cause several problems including distortion of images due to the random placement of the bar, effects to the crystal formation itself or the need to build hardware to remove stir bar shortly after mixing. Instead, an overhead stirring module was designed and mounted onto a mobile X and Z direction unit (again see previously published work[1,2]). This unit can be seen on its moving assembly in Figure 8 and as an exploded view in Figure 9.

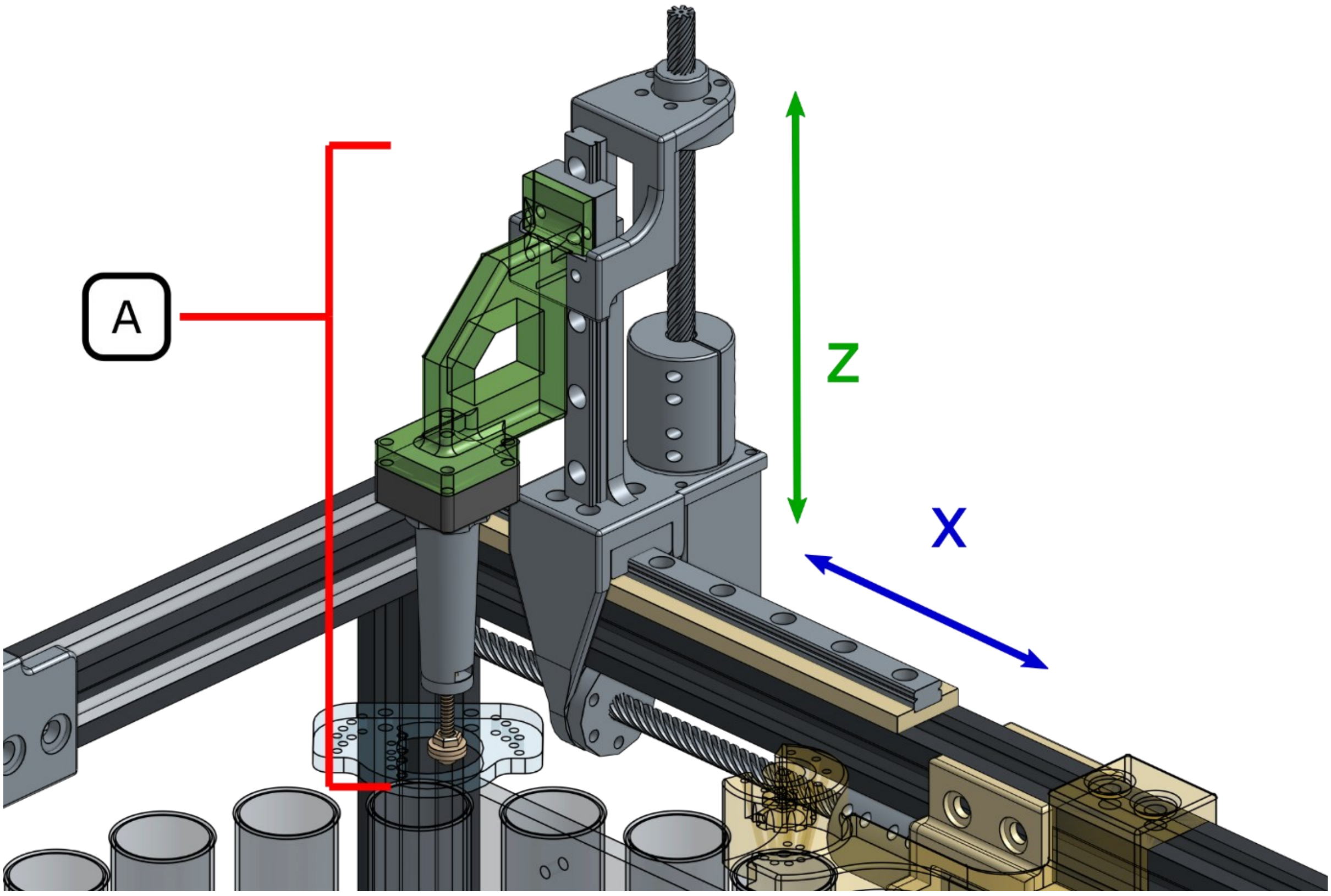

Figure 8. Overhead stirring module on mobile axis**.** (A) Full overhead stirring assembly with X and Z relative motion.

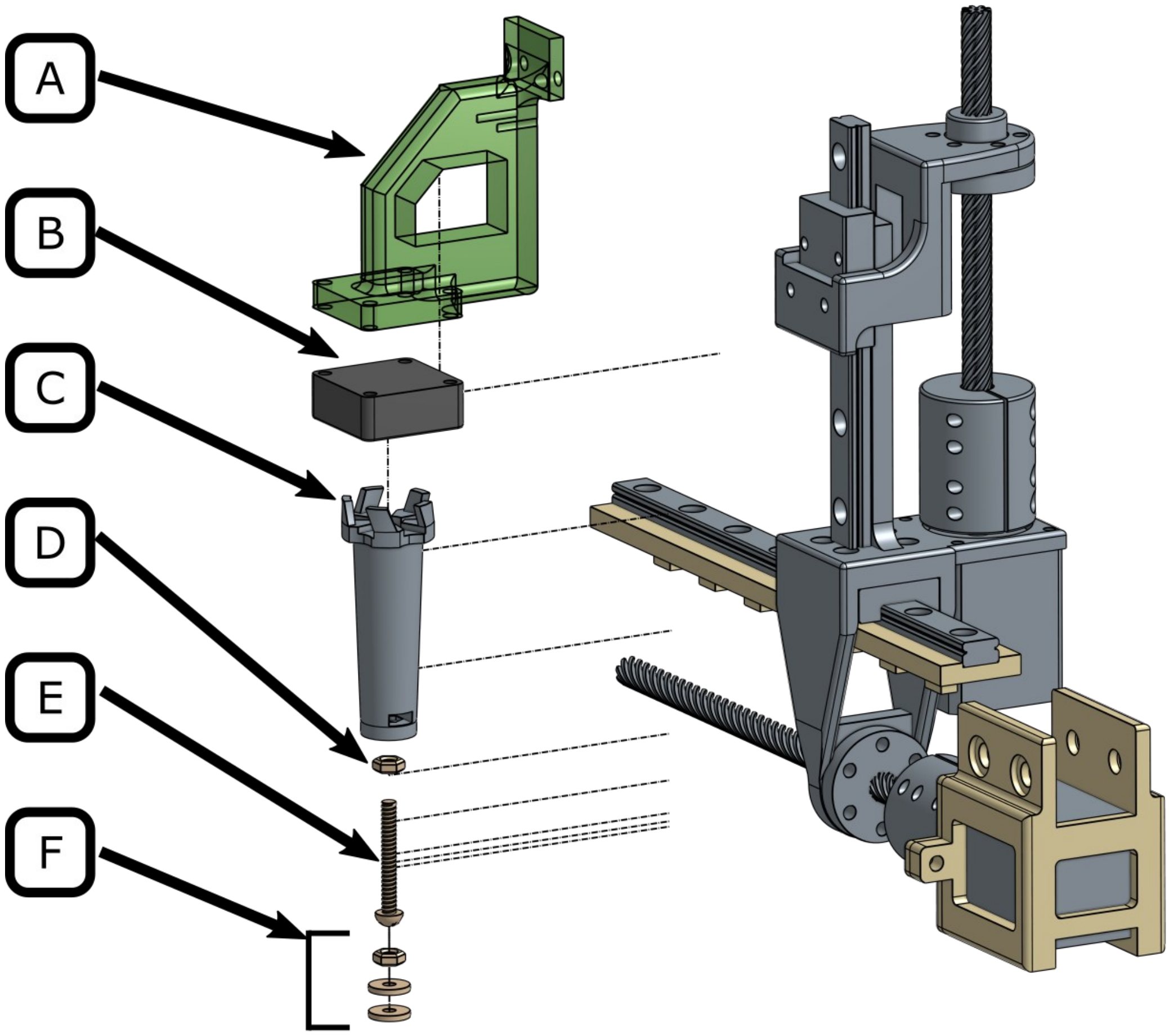

Figure 9 Overhead stirring module exploded view**.** (A) 3D printed, stirring fan mounting unit (connection to the Z axis rail). (B) 25×25mm DC fan usually used for cooling (C) 3D printed rotation shaft (D) Embedded PEEK M3 nut (E) 25mm M3 PEEK screw (F) M3 PEEK nut and 2 spacers (top to bottom).

The fan is controlled by PWM output from the RAMPs board to provide rotation. The two spacers at the base of the stirring unit (Figure 9 (F)) can create a substantial vortex when lowered into a vial. The procedure used to here to ensure adequate stirring without forcing the reaction solution upward on the vial side walls was to initiate the stirring 1 cm above the liquid level, lower the PWM valve to slow the rate to <50 RPM, gently lower the rotating assembly into the solution and cease stirring before homing the Z-axis. This provided a gentle stirring affect whilst ensuring complete mixing. Following the dispensing, the sample is moved anticlockwise by a single turn of the wheel. Stirring occurs immediately following dispensing at this position. Once homed the full overhead stirring assembly is moved in the X-direction (Figure 8) and is lowered into a vial containing acetone, offset from the wheel for cleaning whilst the next sample is being dispensed.

2.2.4 Imaging apparatus

High resolution images of crystallized samples were taken via a HQ Raspberry Pi camera module combined with a Varifocal C-Mount lens (2.8–12mm). The Raspberry Pi itself was used as an imaging server that was constantly awaiting instruction from our Python based experimental software to collect images of fully dried samples. To ensure sufficient and reproducible lighting conditions for images collected, an elaborate camera assembly with diffused lighting from above and below was designed and housed at a single position beneath the vial tray. Samples were rotated above this position and imaged. Figure 10 shows the camera assembly mounted to the base acrylic plate of the MWP both in CAD and real image. Figure 11 shows an exploded view of the camera assembly itself. Figure 12 shows the complete set-up with the camera assembly, Raspberry Pi and above diffused lighting assembly.

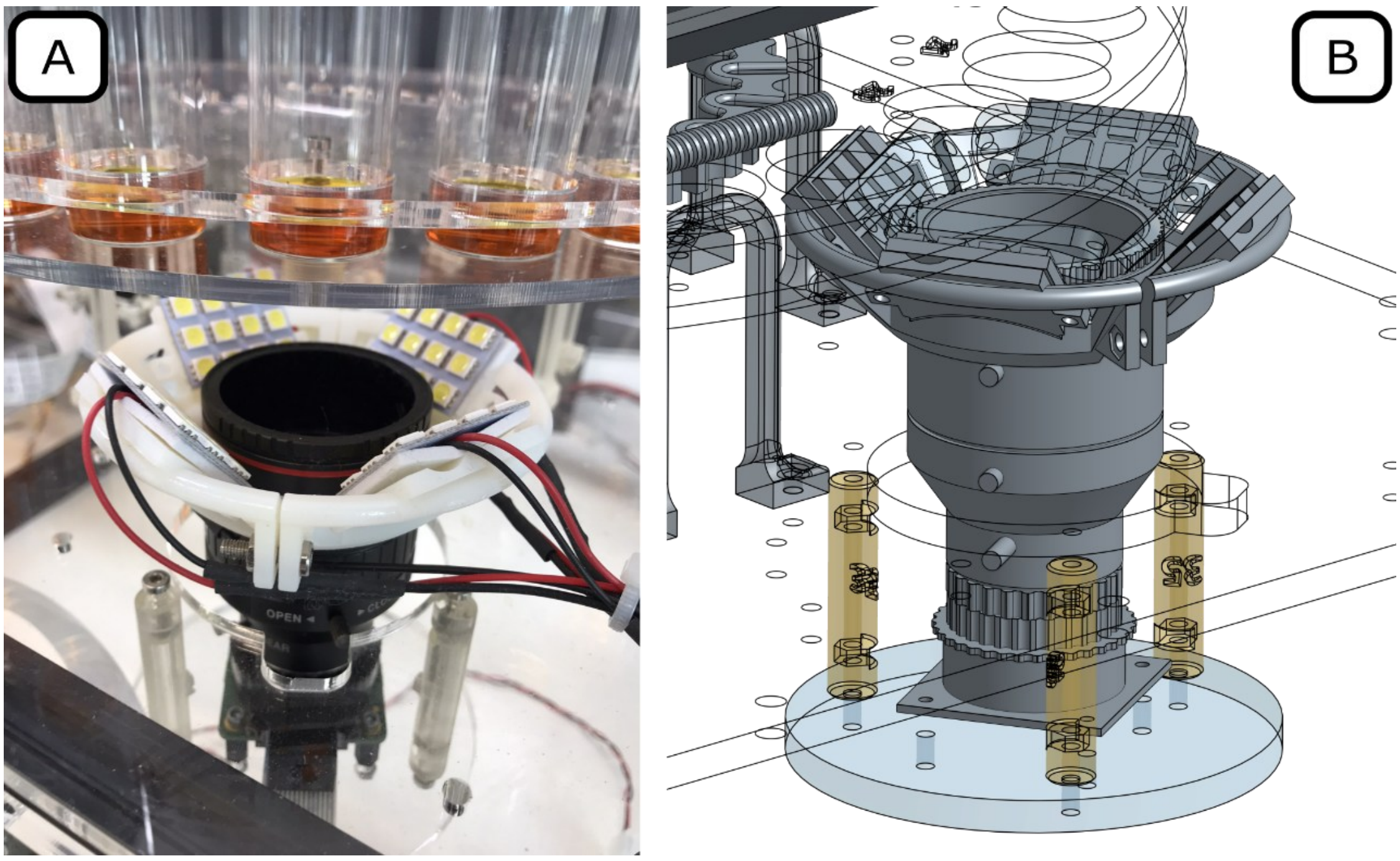

Figure 10 Camera and below vial lighting assembly**.** (A) Real image of camera + below vial lighting assembly bound to the acrylic base plate of the MWP. (B) CAD image of the same assembly for clarity.

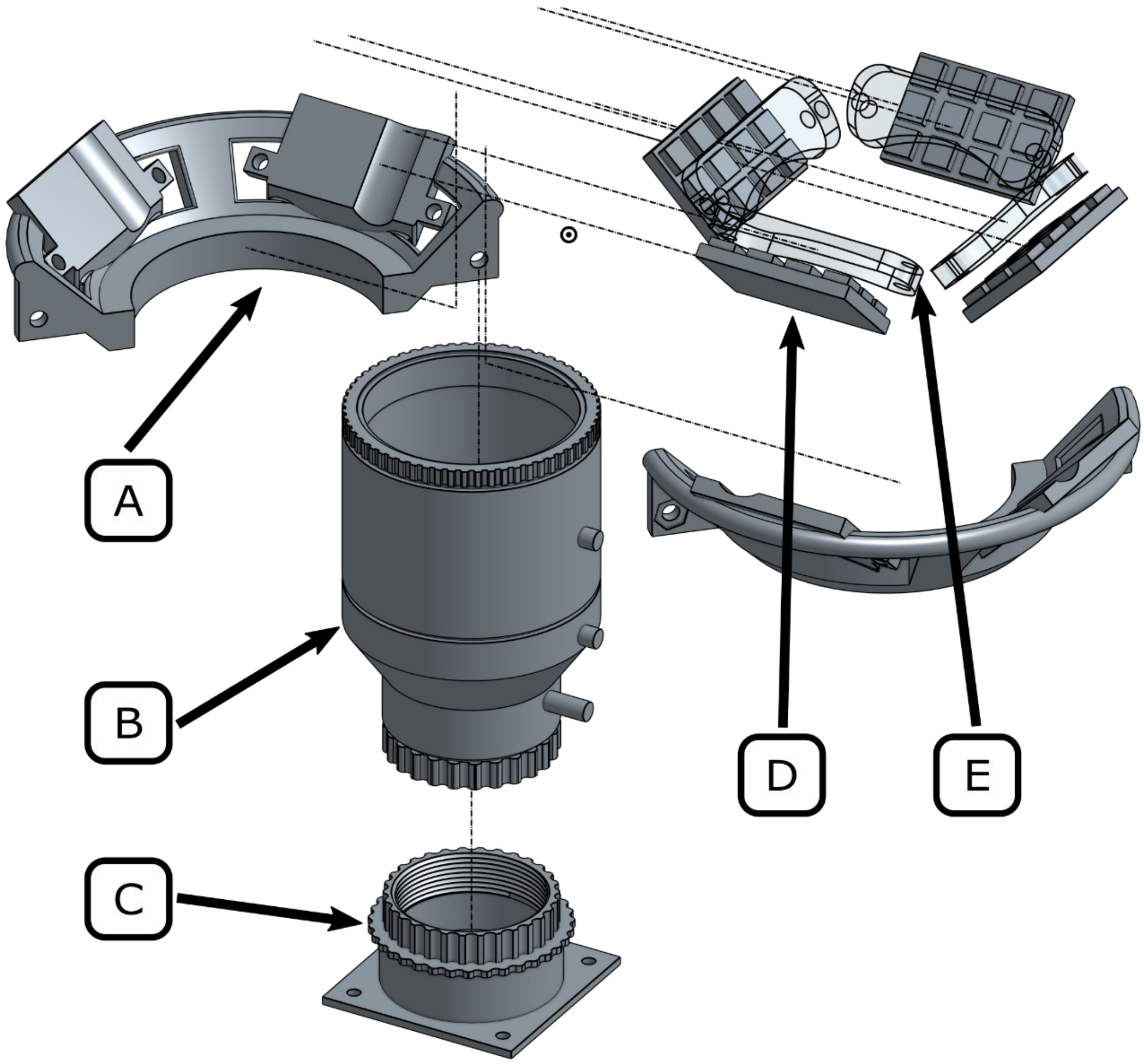

Figure 11. Exploded view of the camera and below vial lighting assembly. (A) One of two semi-circular 3D printed mounts. Each of these unit's house two LED panels directed upward toward the vials (B) C-mount lens for Raspberry Pi high quality (C) Raspberry Pi HQ Camera (D) LED panel (E) Diffused acrylic light cover.

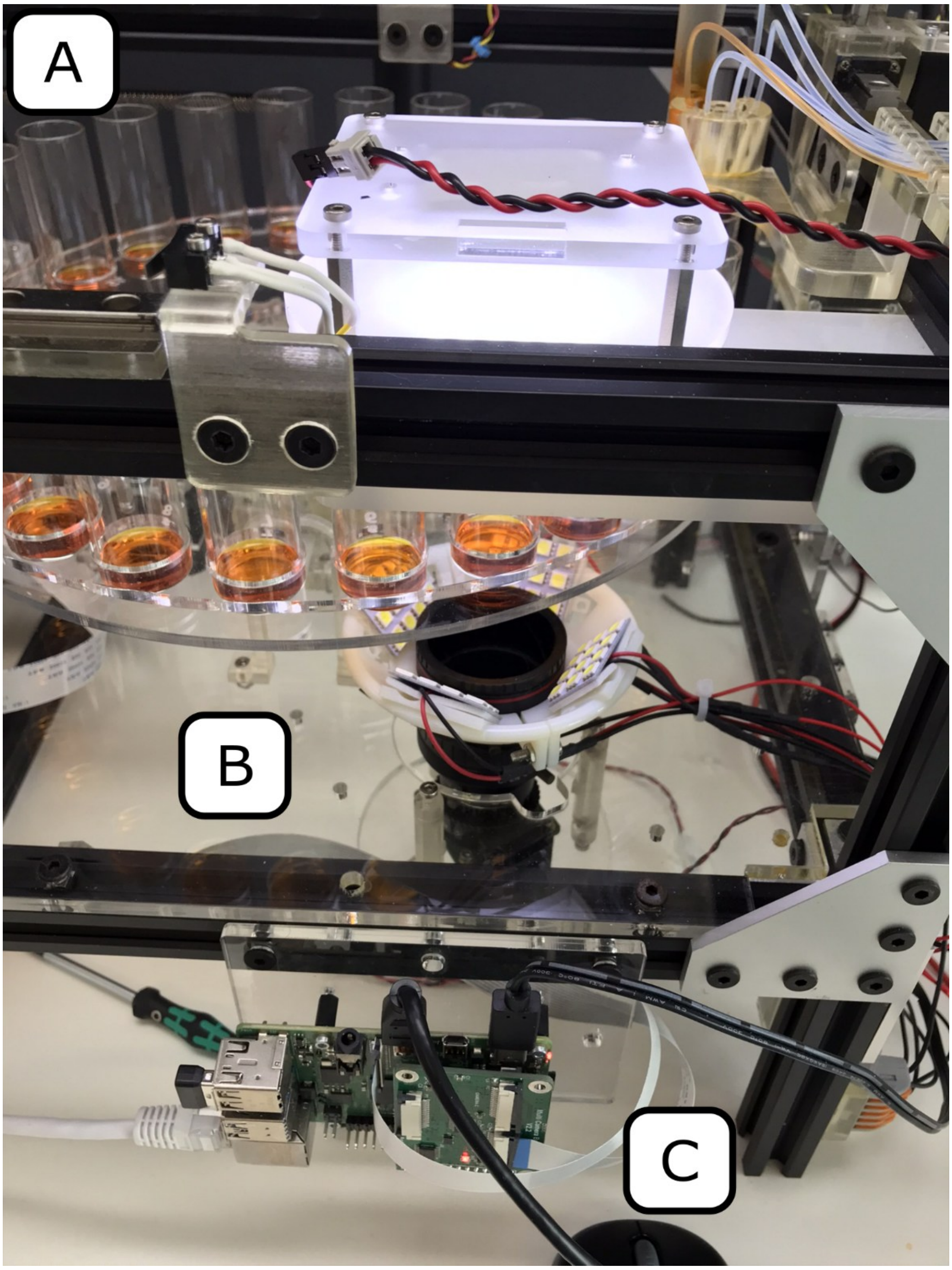

Figure 12. Actual camera/light/Pi assembled on the system. (A) Two layers of frosted acrylic with LED panel in between for lighting above vials. (B) HQ camera + lens/lighting mounted below vials on the acrylic base plate (C) Raspberry Pi (imaging server) mounted to platform frame.

2.2.5 Combined liquid handling and imaging set-up

The previous three sections complete the area of the platform concerned with sample preparation and imaging. This complete sent be seen in Figure 13.

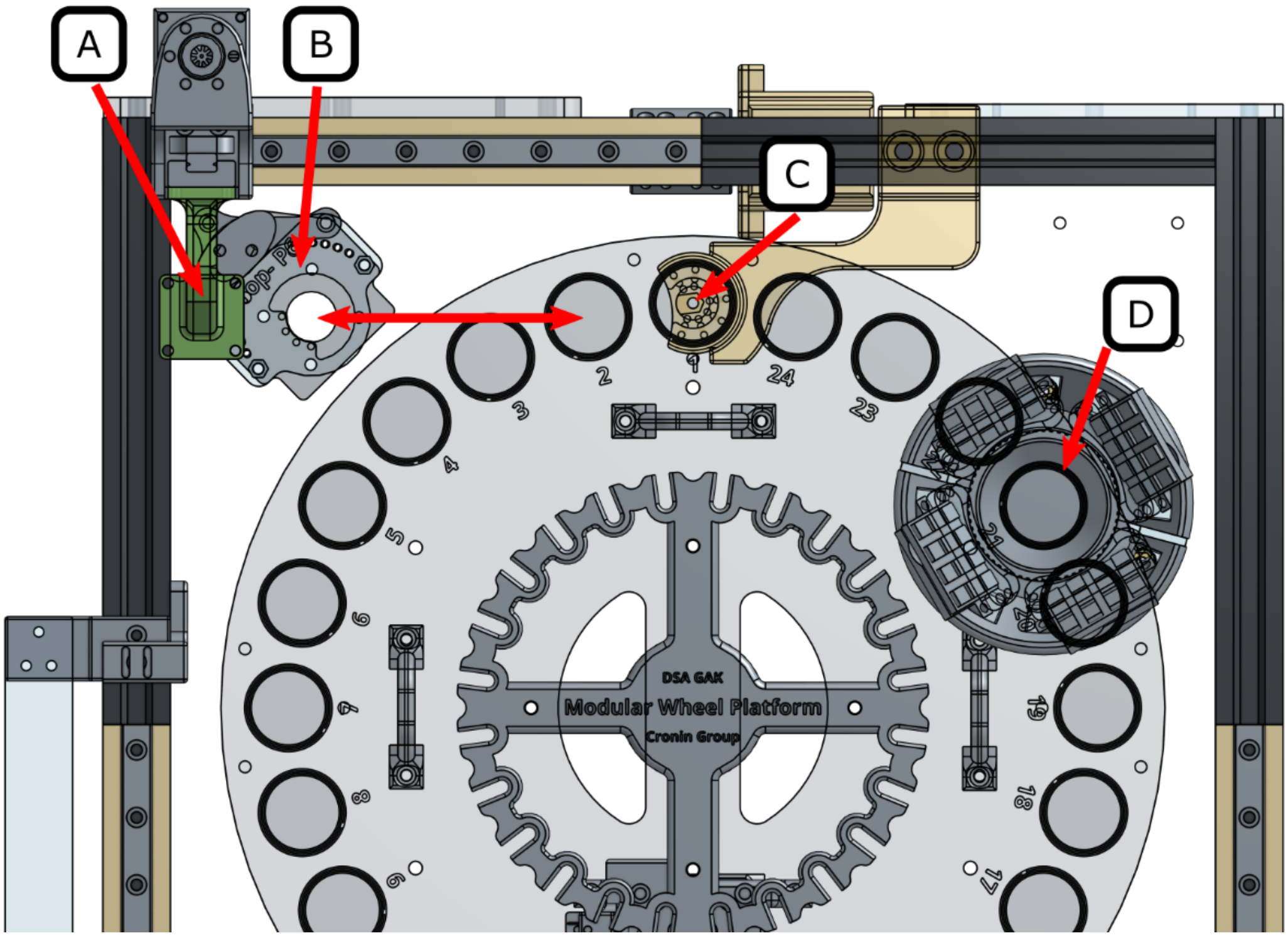

Figure 13. Completed sample preparation and imaging apparatus. (A) Stirring assembly, (B) Acetone washing station, double headed arrow showing the motion between this area and the sample 2 position where stirring occurs, (C) Dispening at position 1, (D) Under vial tray camera assembly.

2.2.6 Vial handling

To move samples from the sample preparation wheel to the storage area and vice versa, a vial handling apparatus was designed. To begin, an X-Y motion axis system was constructed using similar hardware to that seen in the overhead stirring module but on a larger scale. The Y-axis motion was driven by two parallel Nema lead screw motors (48mm stack, 1.68A, 300 mm lead length) bound to 395 mm NSK linear rails (NSK PU S(eries, P1U090600SKN-PCT, 9mm width) by a custom 3D print and two linear guide carriages (NSK PAU09TRS, PU). Endstop switches were again used for positioning feedback. A cross-section view of the Y-axis set-up can be seen in Figure 14.

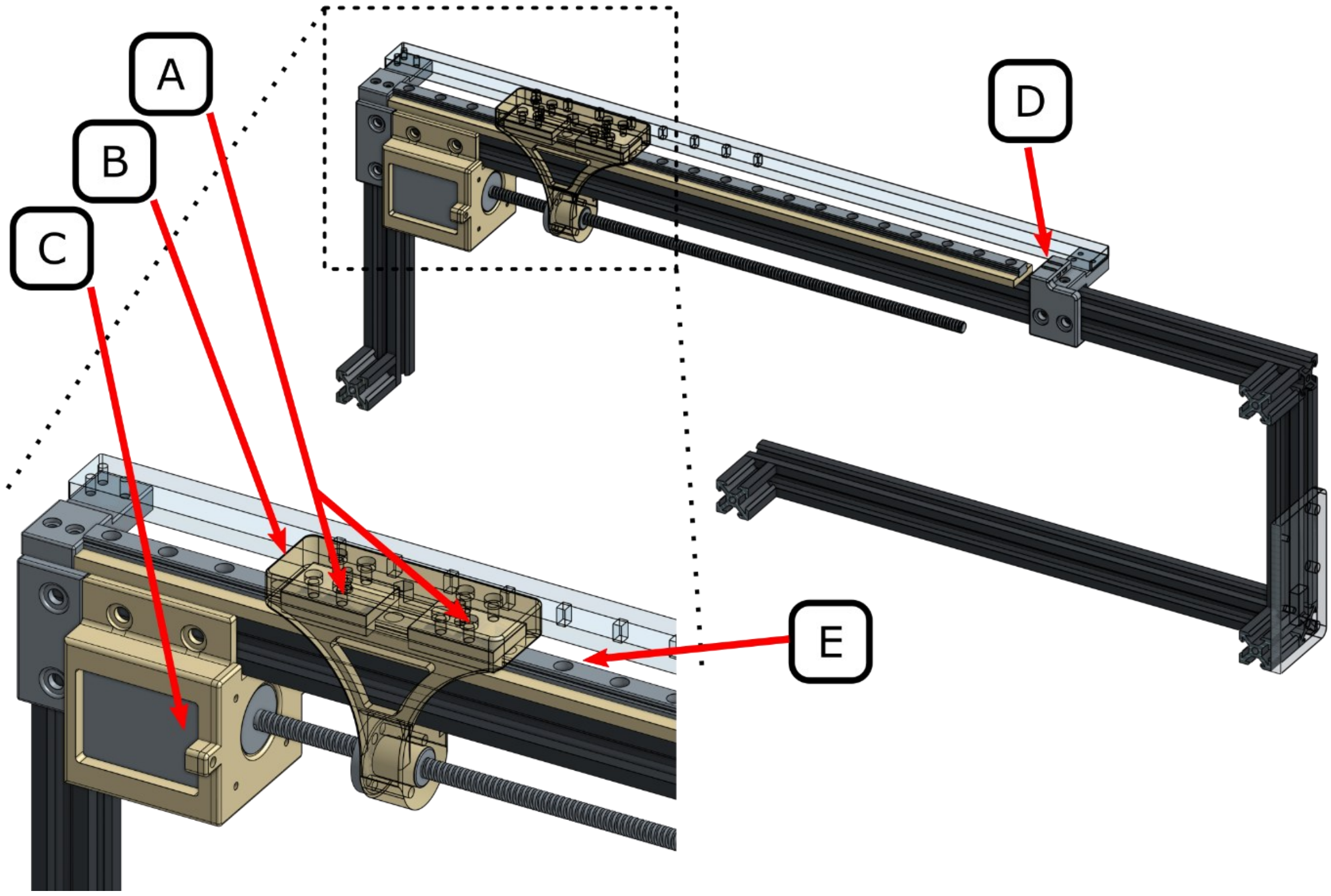

Figure 14. Cross section of Y-axis drive hardware. (A) Two NSK PAU09TRS linear guide carriage internally bound to (B) 3D printed joining part from linear carriages/rail to (C) lead screw nema17 motor. (D) 3D printed endstop mount (E) NSK P1U090600SKN-PCT 9 mm linear rail.

The X-axis assembly mounts to the printed parts labelled (B) in Figure 14. This assembly uses identical hardware components to the Y-axis with the exception of the motor used, in this case a nema14 lead screw motor (48 stack, 0.91A, 250 mm lead screw length). The assembly moves a general use 3D printed mounting plate along the linear rail. This plate (Figure 15 (D)) doubles as the link between lead motor, linear guide and rail and as the mounting area for different

functionalities a user might need for the workflow. In the case of this system, an 8 mm acrylic base plate housing a gripping assembly is used.

The X-axis assembly is shown in Figure 15.

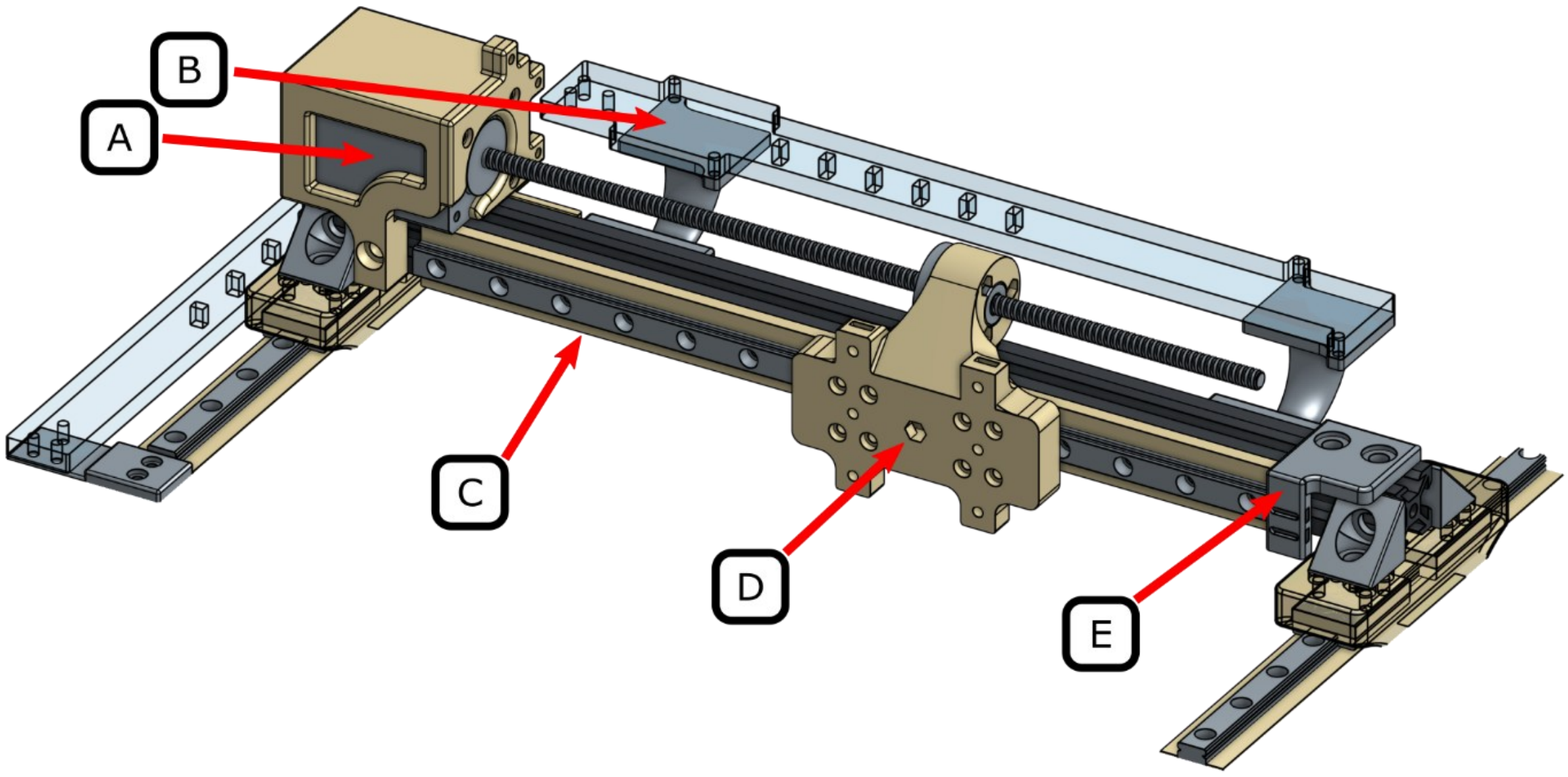

Figure 15. X-axis assembly. (A) Nema14 250 mm lead screw motor (B) Cable chain mount (C) NSK P1U090600SKN-PCT 9 mm linear rail (D) 3D printed mounting plate with two embedded NSK PAU09TRS linear guide carriages (E) 3D printed endstop mount.

The gripping mechanism is again an assembly of commercially available linear motion hardware and custom 3D printed parts. A number of challenges needed to be considered in the design of this module:

1. The confined space available
2. The need for a converging linear motion due to the hexagonal close packed nature of the vials across the system i.e. a typical geared mechanism is arm converging by a mirrored circular motion would have impacted adjacent vials and failed.
3. Gripping strength as the smaller the motor, the weaker the ability to apply force.

The complete assembly can be seen in Figure 16 with the main components labelled.

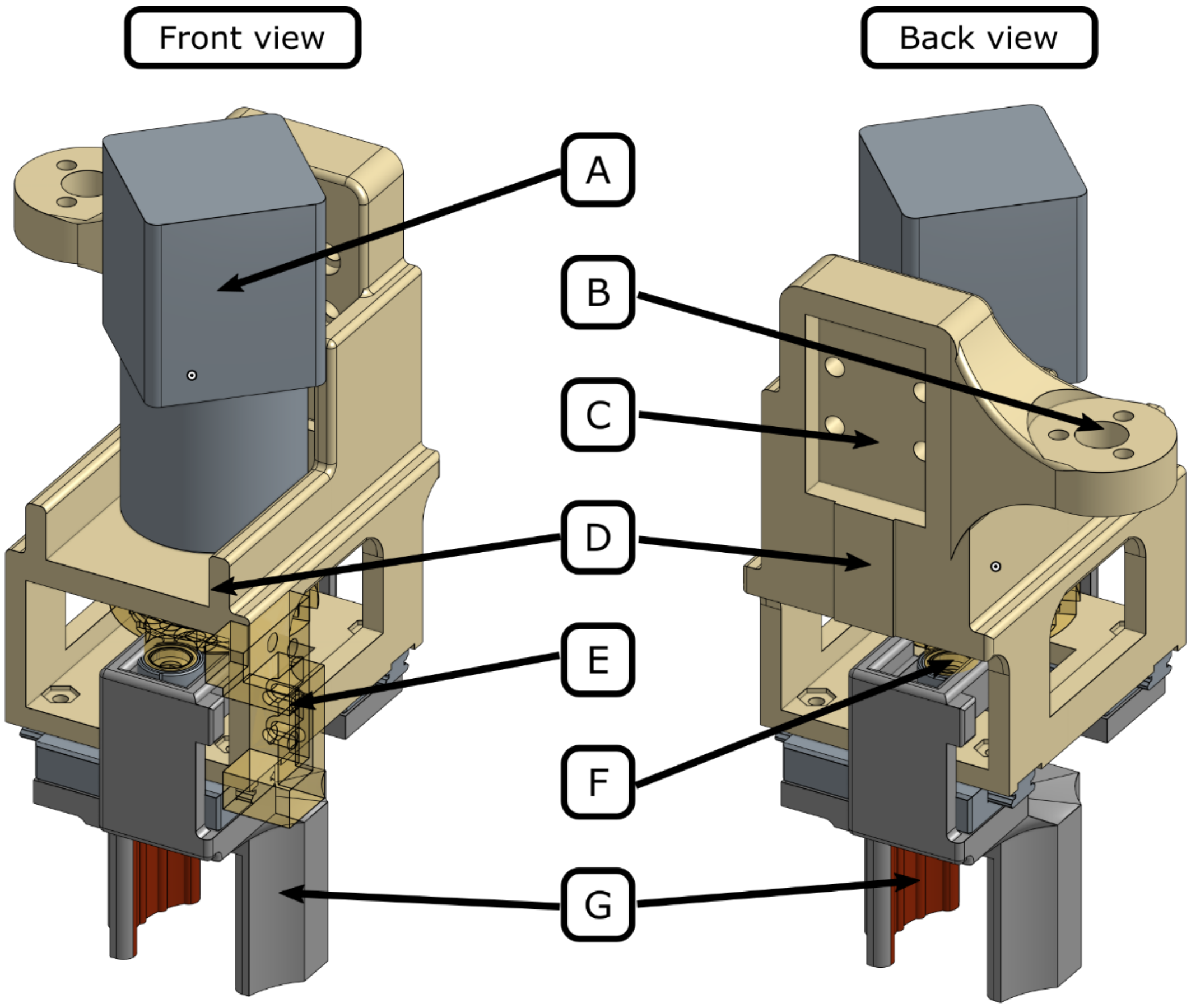

Figure 16. Gripper assembly overview front and back**.** (A) Geared nema11 stepper motor (1:5 ratio) (B) Printed joining position to Z-motor lead screw nut (C) connection outline for NSK PAU09TRS linear guide carriage (D) Single piece 3D printed main body (E) 3D printed endstop positioning switch mount (F) Contact point bearing from motor to gripper arm 3D prints (G) Gripper arm 3D prints.

The issue around gripping strength was solved by using the geared nema11 stepper motor (Figure 16 (A)). This motor provides significantly higher torque than a normal nema11 motor and great accuracy due to the reduced rotation per motor step.

A Pololu aluminium mounting hub is attached on the 6mm shaft of this geared nema11 motor. Two symmetrical 3D printed parts are screwed to the hub and a 10 mm OD, 6 mm ID bearing is press fit to the arm like structure on both (Figure 17). These parts move in a cylindrical motion, driven by the motor.

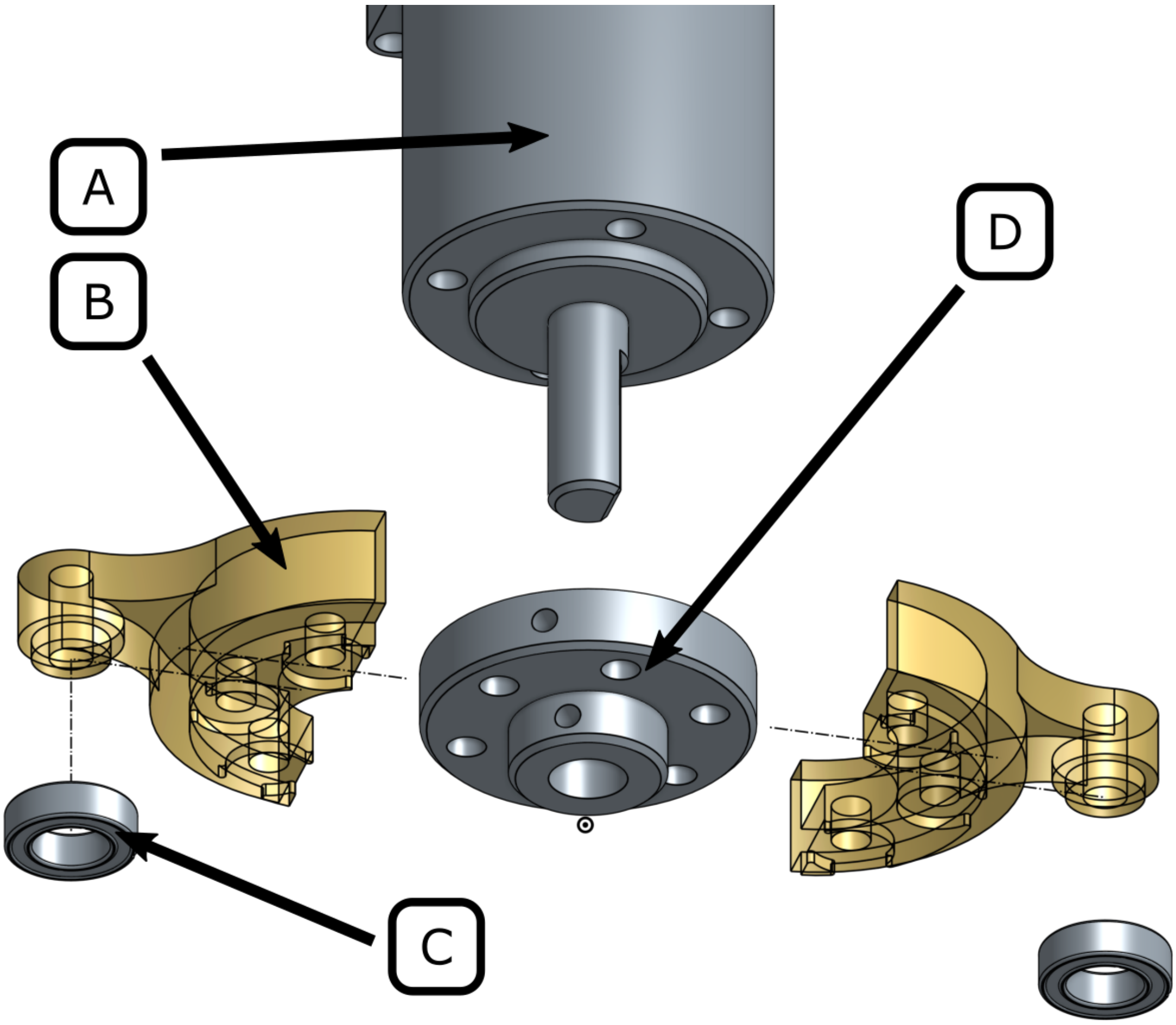

Figure 17. Gripper cylindrical motion components. (A) Nema11 geared motor with 6 mm shaft (B) 3D printed attachments for hub and bearings (C) 10 mm OD 6 mm ID steel bearing (D) Pololu aluminium mounting hub.

The cylindrical motion provided by this assembly is converted to a linear motion by the two bearings making contact and driving the gripper arms that are each bound to miniature linear rails and guide assemblies.

3. Crystal Vision and Analysis

3.1 Training MARCO dataset clustering

Training images were obtained from the MARCO database as jpg files: https://marco.ccr.buffalo.edu/data/train-jpg/. Datasets train-00001-of-00407 and train-00002-of-00407 were labelled using the tags ‘crystal’, ‘cluster’ and ‘precipitate’. The configuration file has json formatting and is shown below.


```json
{
    "object": "crystal",
    "version": "v2",
    "classes": [
        "single",
        "cluster",
        "powder"
    ],
    "root_directory": "~/marco",
    "train": {
        "max_iter": 1000,
        "base_lr": 0.00080,
        "eval_period": 50,
        "split_rate": 0.8
    }
}
```


Figure 18. Configuration file for training of generic crystal detection model

The loss per iteration for rpn location and rpn, class, mask, class and bounding box as well as total loss and validation loss are shown below. The training was ended early at epoch 1000 in order to avoid overfitting.

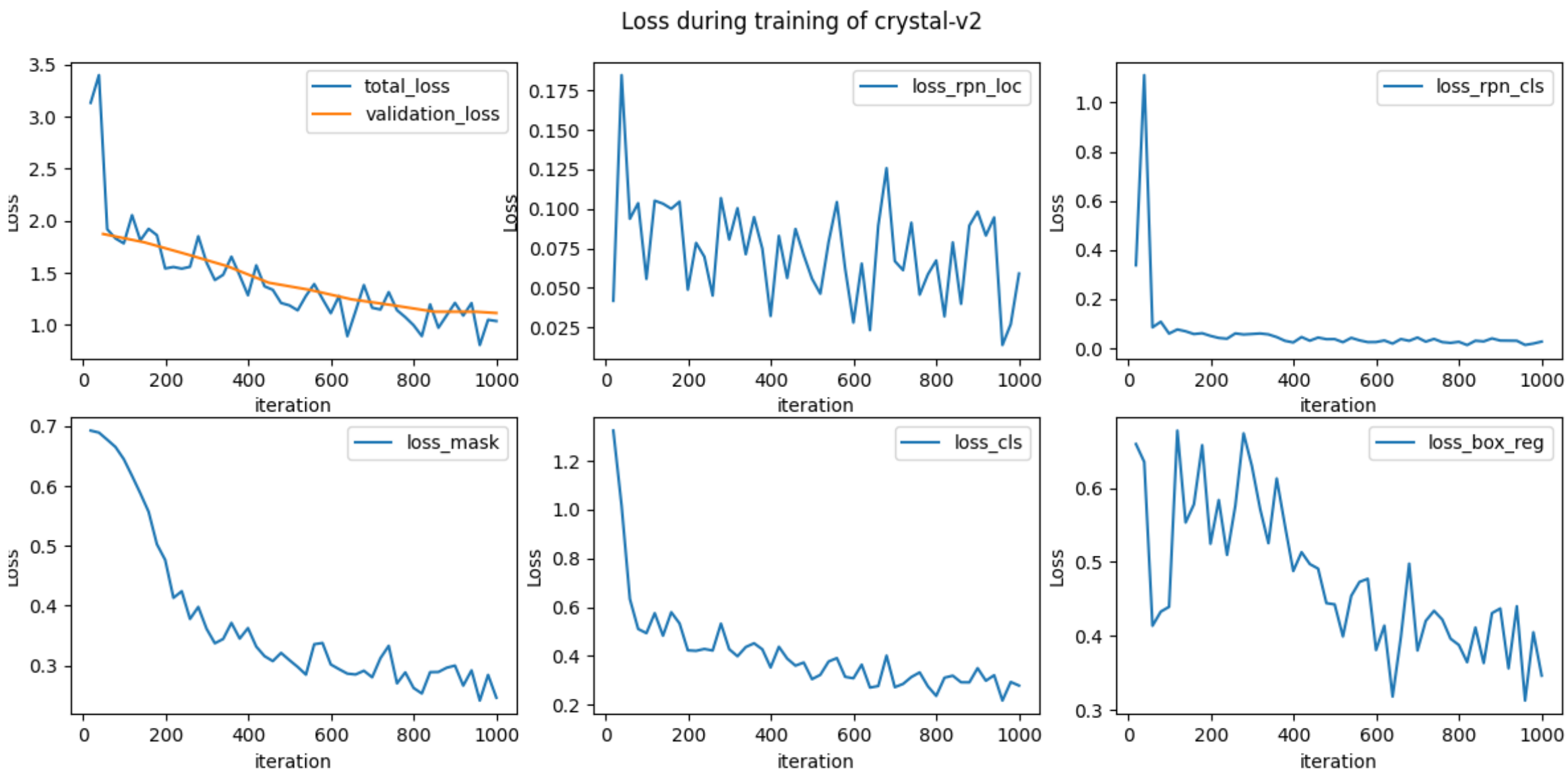

Figure 19. Training metrics for generic crystal detection tl: total and validation loss, tm: rpn location loss, tr: rpn class loss, bl: mask loss, bm: class loss, br: bounding box loss

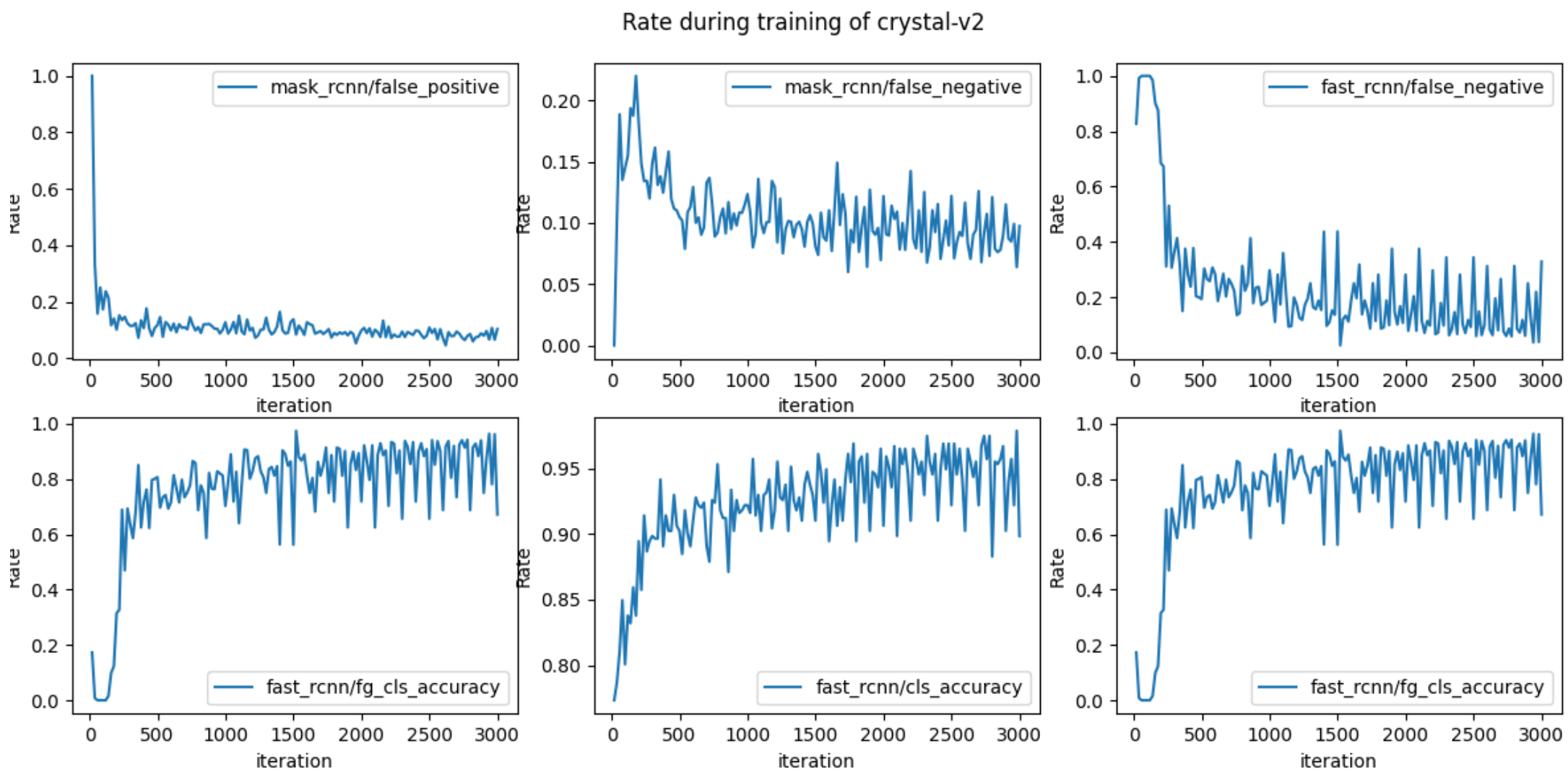

Figure 20. Training metrics for generic crystal detection tl: mask rcnn false positive rate, tm: mask rcnn false negative rate, tr: fast rcnn false negative rate, bl: fast rcnn foreground class accuracy, bm: fast rcnn class accuracy, br: fast rcnn foreground class accuracy

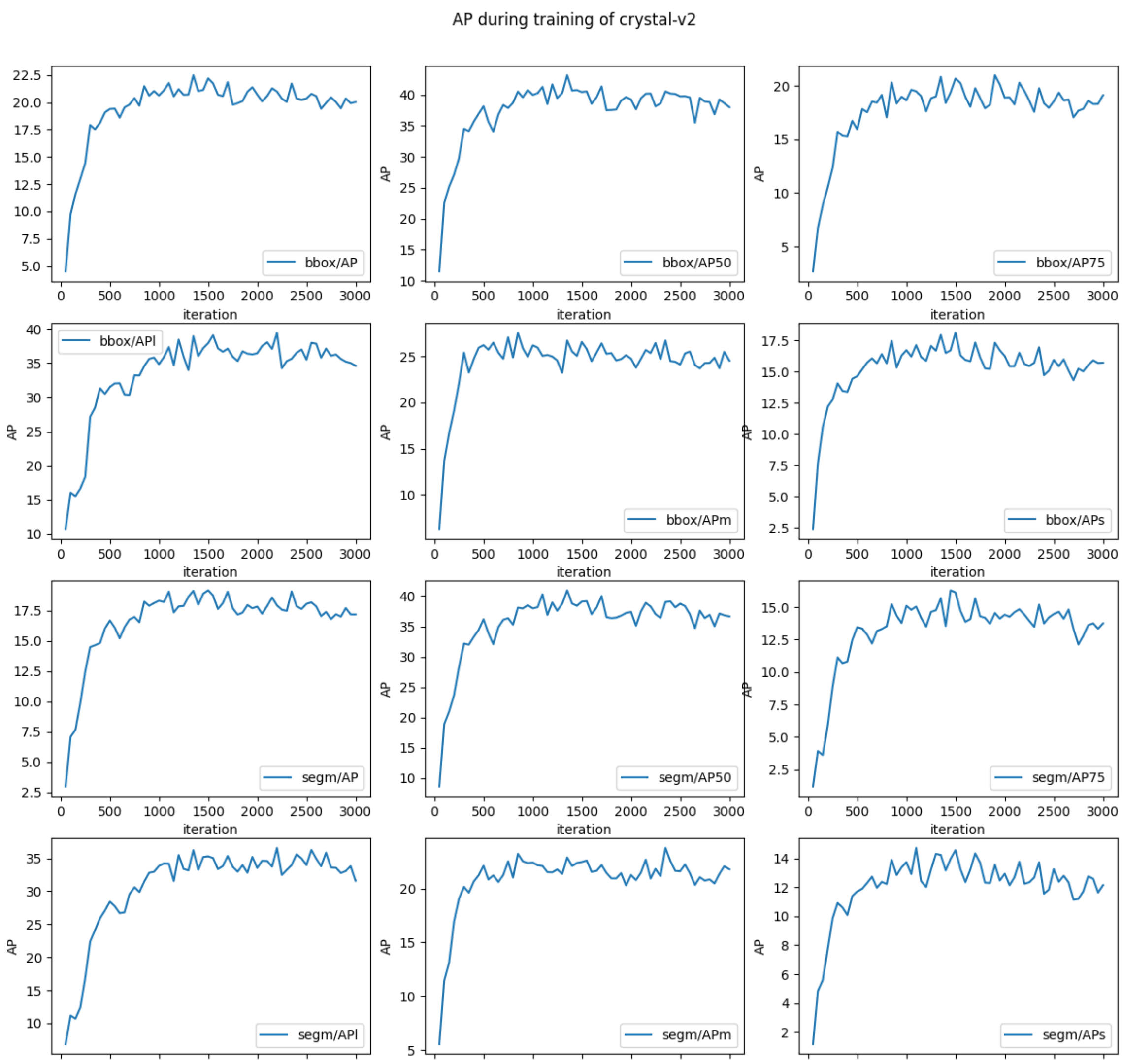

Figure 21. Training metrics for generic crystal detection. Average precision values for the bounding boxes and segmentations

eval	AP	AP50	AP75	APs	APm	APl
bbox	16.63776	30.25702	16.1557	11.69802	18.95614	37.71491
segm	14.41656	30.11181	10.7515	7.178422	19.67313	36.91637

Table 1. Average Precision of trained crystal detection model.

Applying the classifier on images from (MARCO: train-00004-of-000407-dir) using a threshold score of 0.4 results in the following detections:

Id/description	Original	Crystal detector	Aggregation detector
224114.jpeg / Large discrete single crystals.	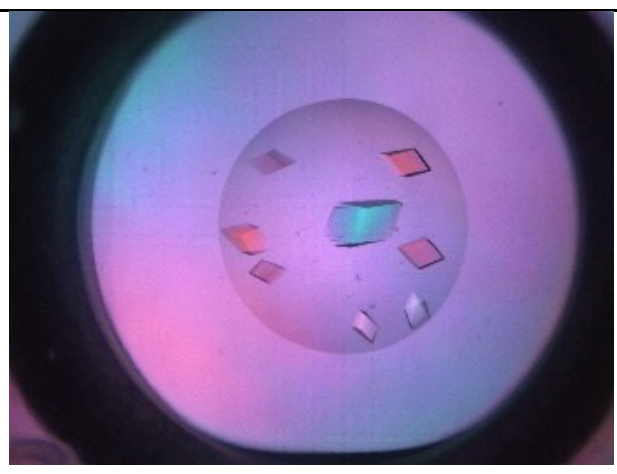	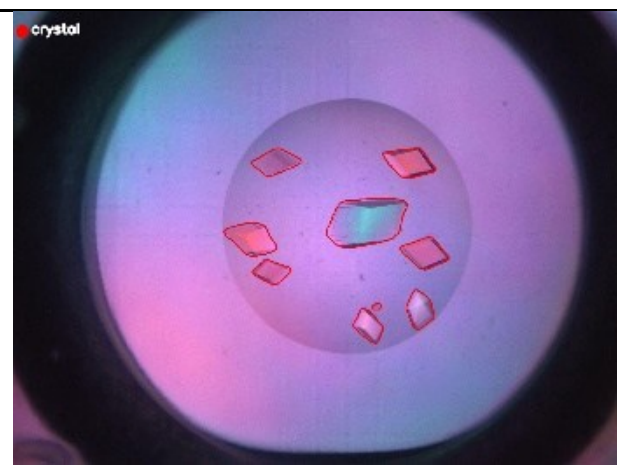	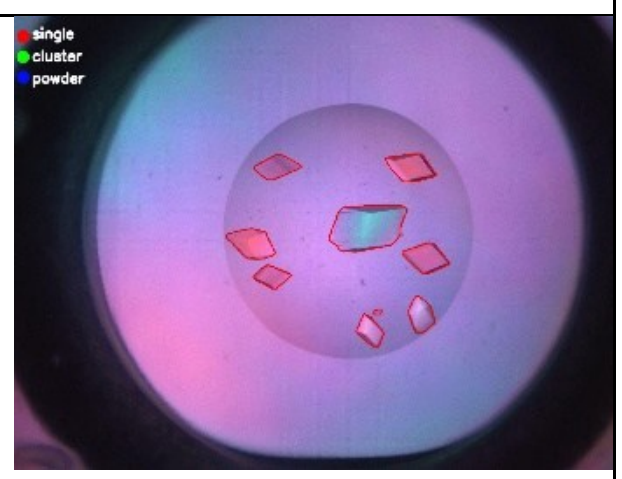
45093.jpeg / Small discrete single crystals. Some grouping of adjacent crystals.	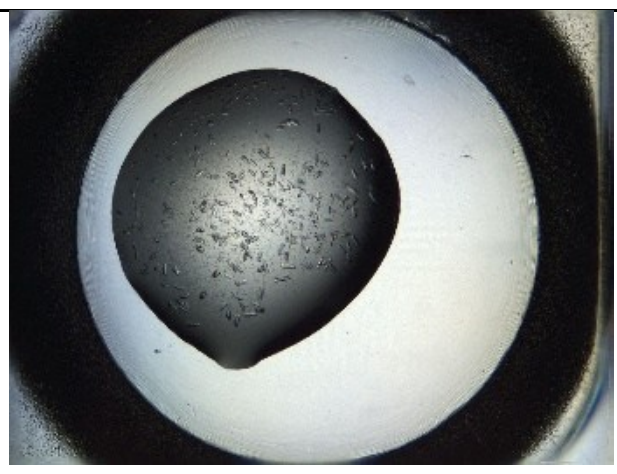	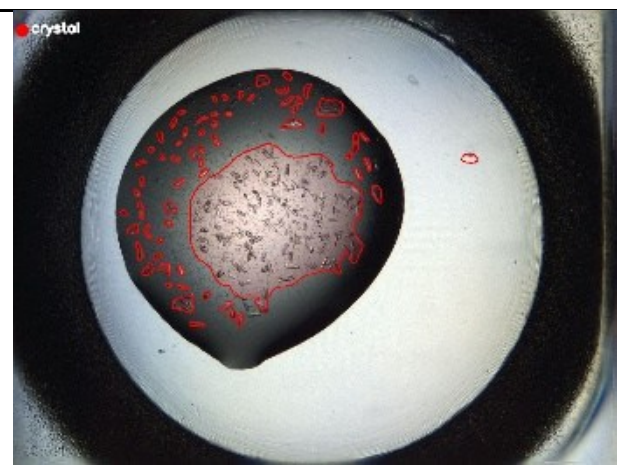	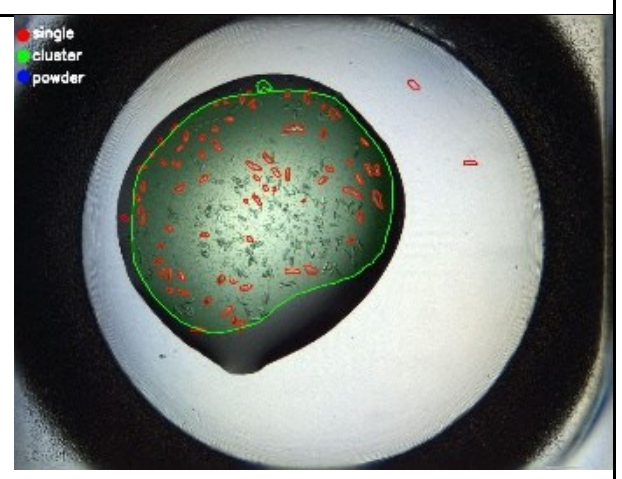
358838.jpeg / Small continuous single crystals. Crystals identified as contiguous area	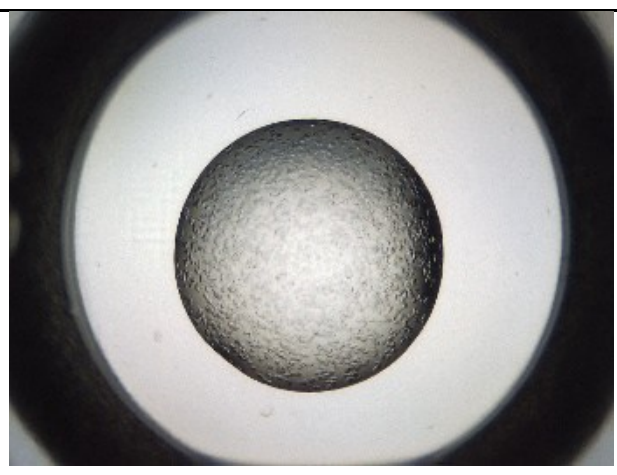	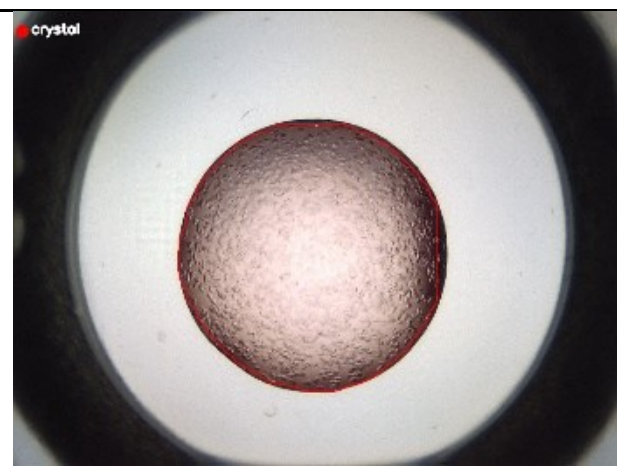	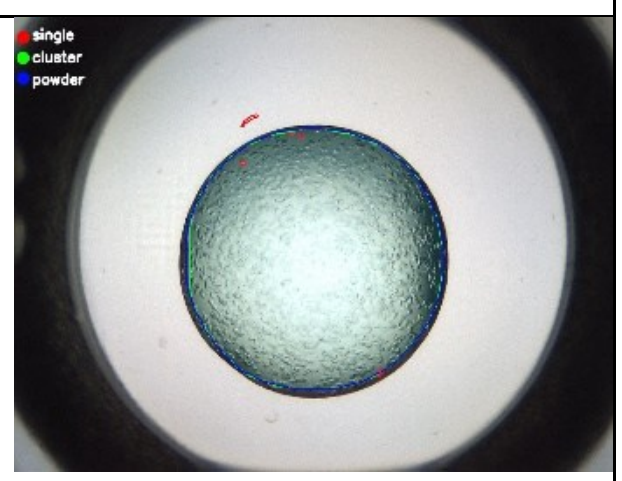
185890.jpeg / Large discrete crystal clusters.	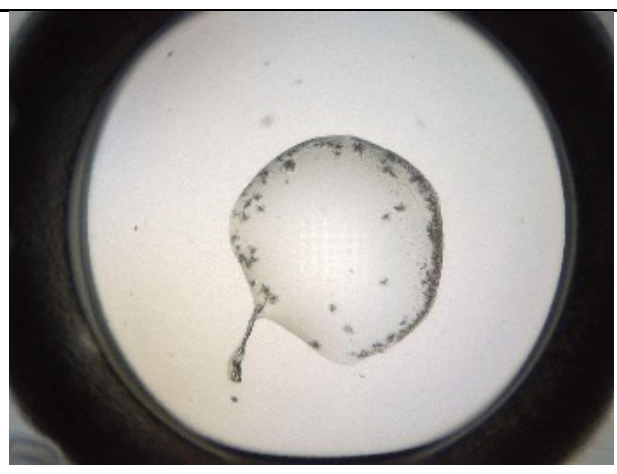	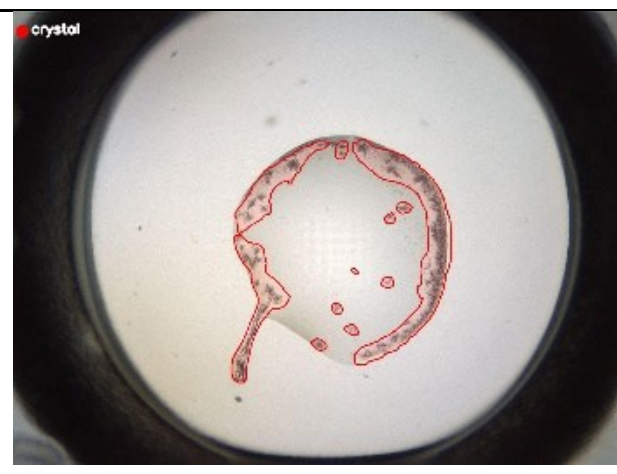	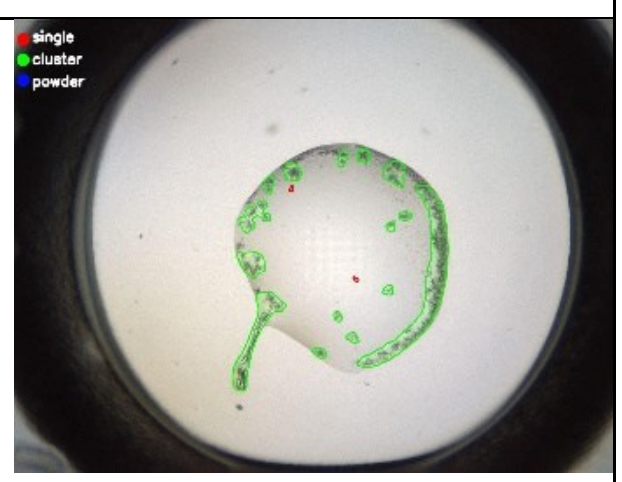

481031.jpeg / Many clustered crystals. Crystals identified as contiguous area	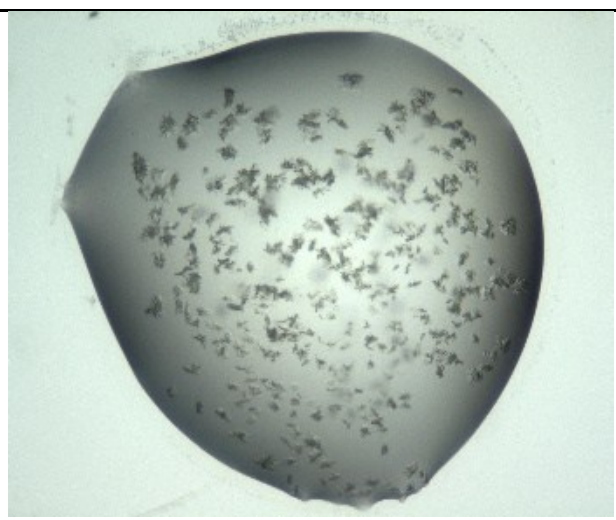	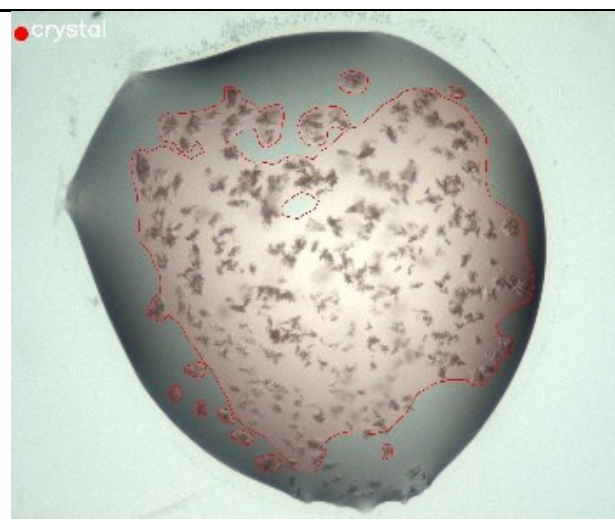	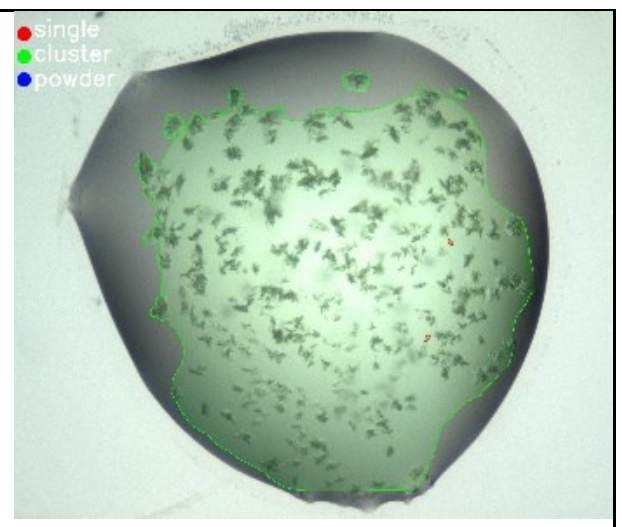

Table 2. Examples of detections using the MARCO trained models. Left is the original image. Centre shows the detections by the generic crystal detector in red. Large single crystals are detected tightly around their perimeter. Smaller clusters of overlapping crystals are identified as an area which includes some solvent. Right shows the detections by the specific classifier, with single crystals labelled in red, clusters in green and powder in blue. Large single crystals are classified tightly around their perimeter in red. Smaller clusters are identified as an area that includes some solvent in green, with some single crystals identified within the area in red.

3.2 Training ROY dataset-polymorph

Training images were obtained from the from crystals that had been produced on the platform and were labelled using the tags 'ON', 'YN', 'OP', 'Y', 'R', 'ORP'. The configuration file has json formatting and is shown below.

```
{
    "object": "polymorph",
    "version": "v1",
    "classes": [
        "ON",
        "YN",
        "OP",
        "Y",
        "R",
        "ORP"
    ],
    "root_directory": "~/ROY",
    "train": {
        "max_iter": 2000,
        "base_lr": 0.00080,
        "eval_period": 50,
        "split_rate": 0.9
    }
}
```

Figure 22. Configuration file for training of ROY polymorph crystal detection model

The loss per iteration for rpn location and rpn, class, mask, class and bounding box as well as total loss and validation loss are shown below. The training was ended early at epoch 1000 in order to avoid overfitting, due

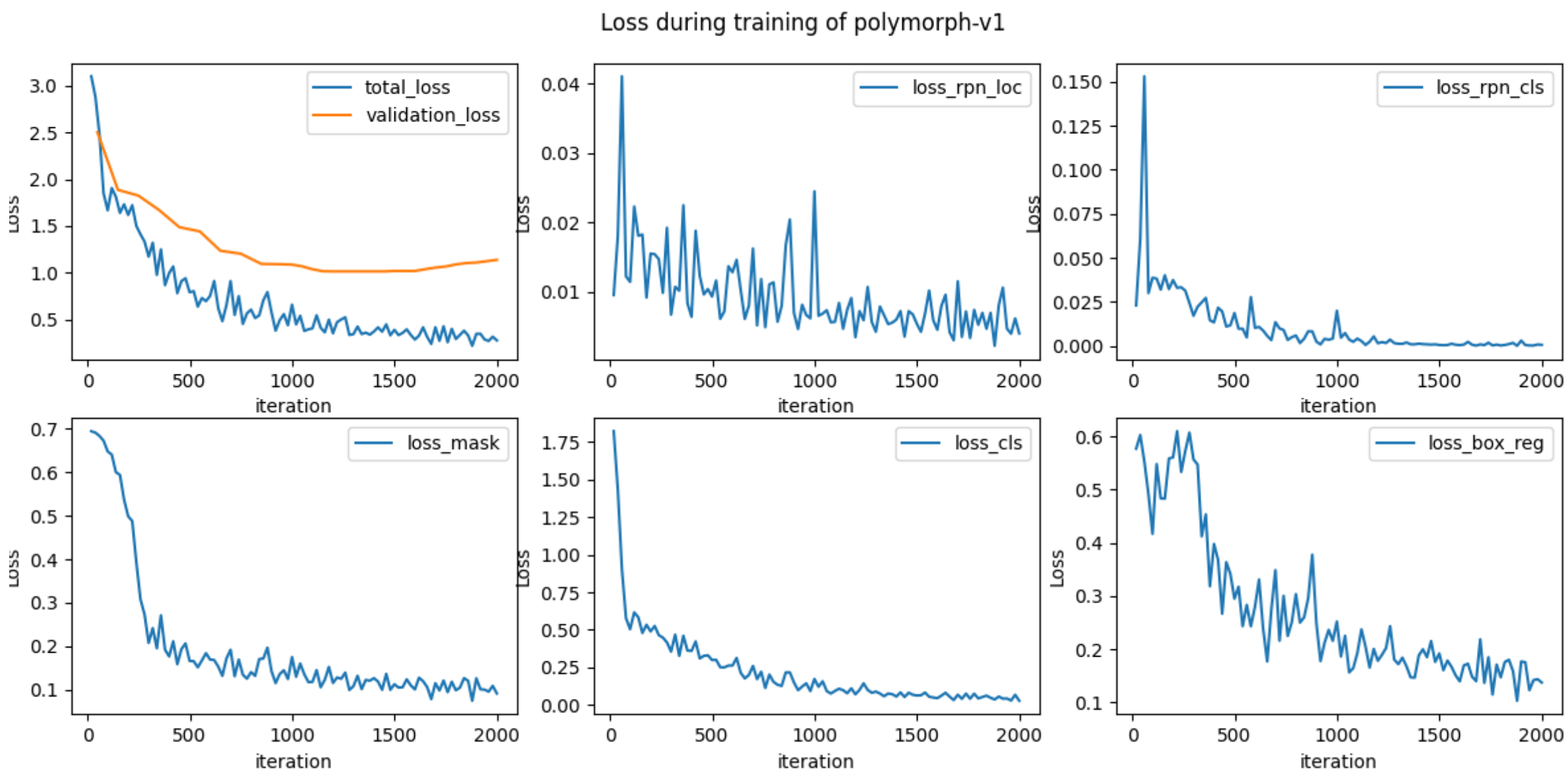

Figure 23 Training metrics for ROY polymorph detection. tl: total and validation loss, tm: rpn location loss, tr: rpn class loss, bl: mask loss, bm: class loss, br: bounding box loss

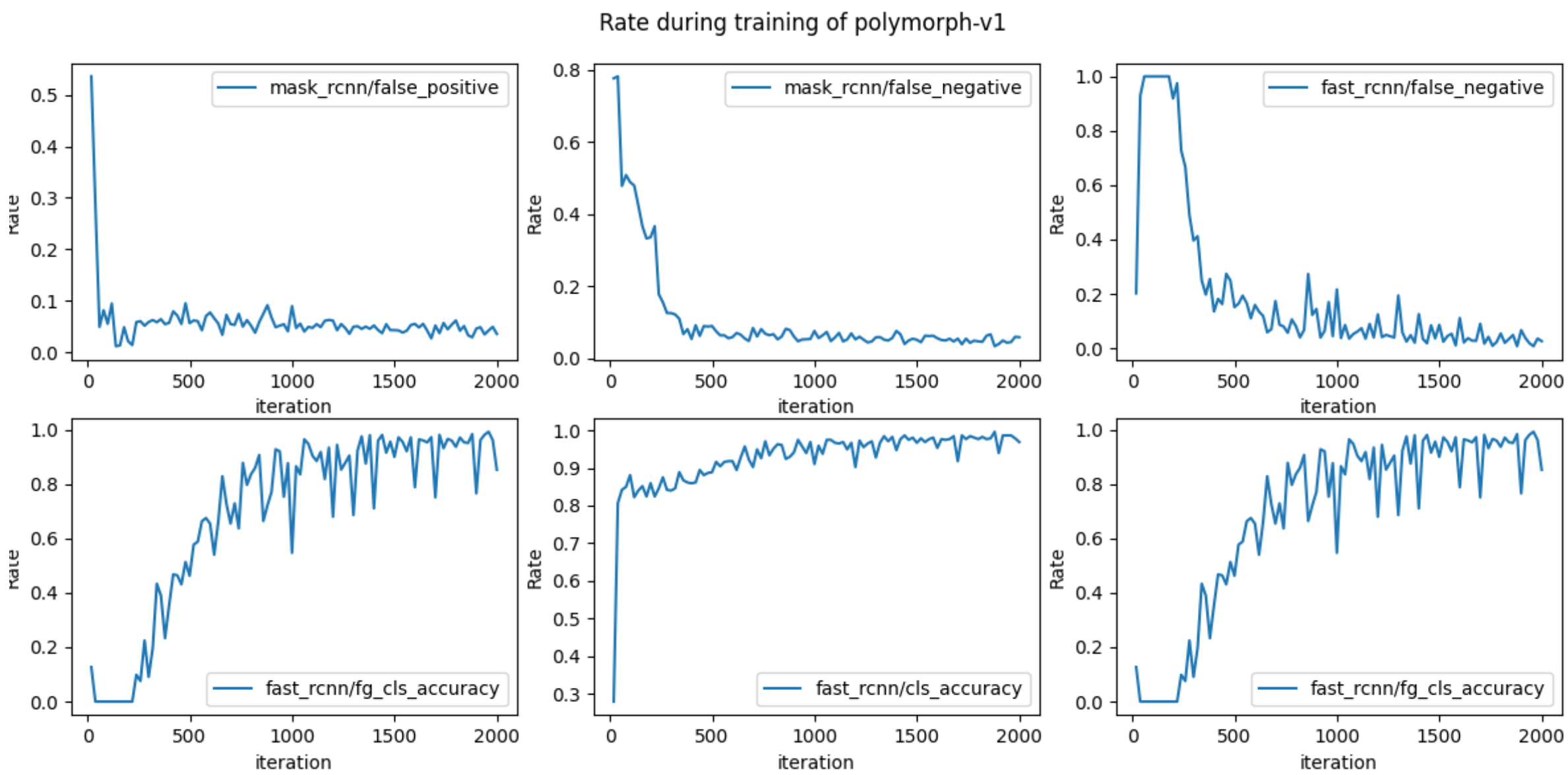

Figure24. Training metrics for ROY polymorph detection tl: mask rcnn false positive rate, tm: mask rcnn false negative rate, tr: fast rcnn false negative rate, bl: fast rcnn foreground class accuracy, bm: fast rcnn class accuracy, br: fast rcnn foreground class accuracy

AP during training of polymorph-v1

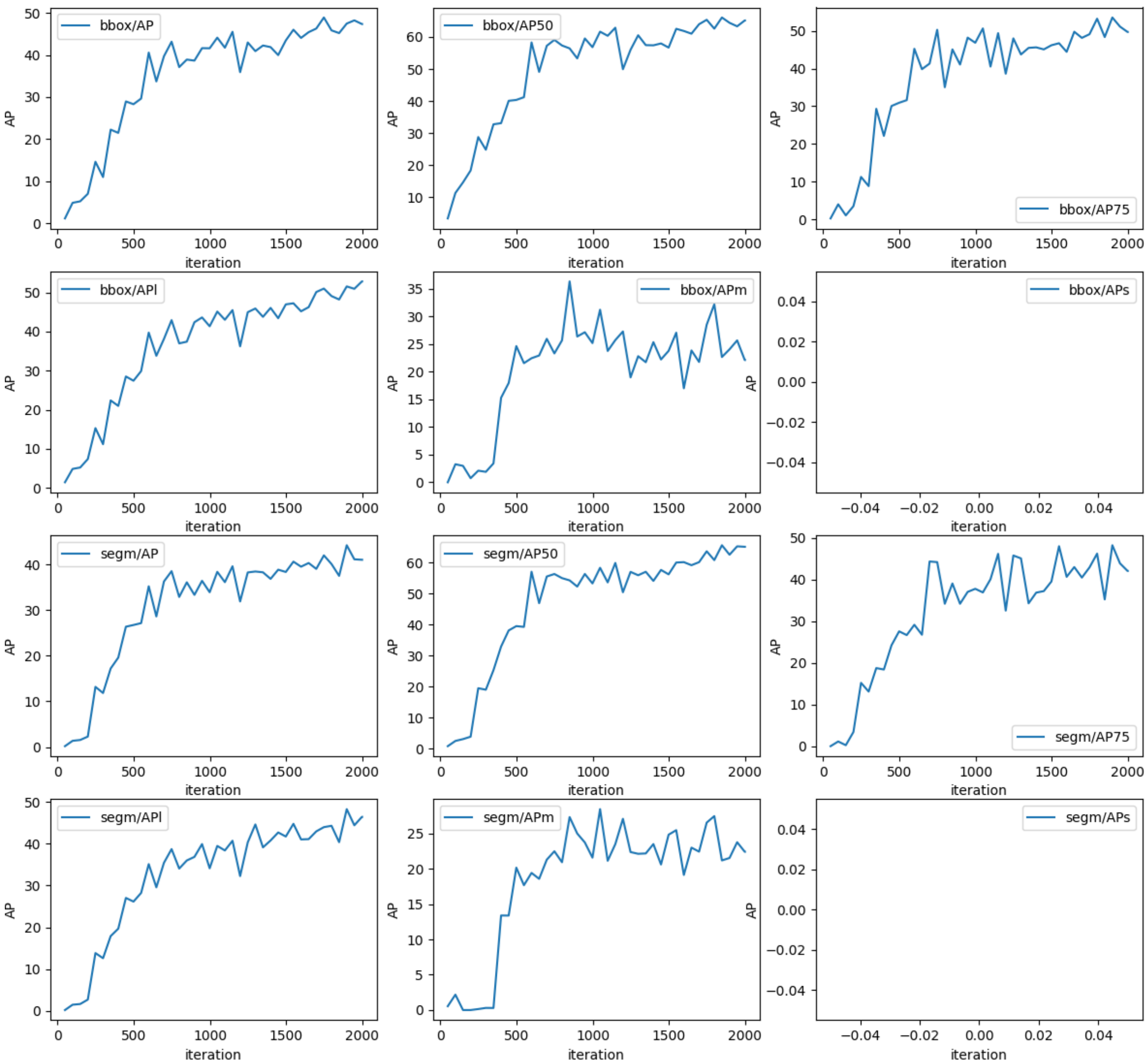

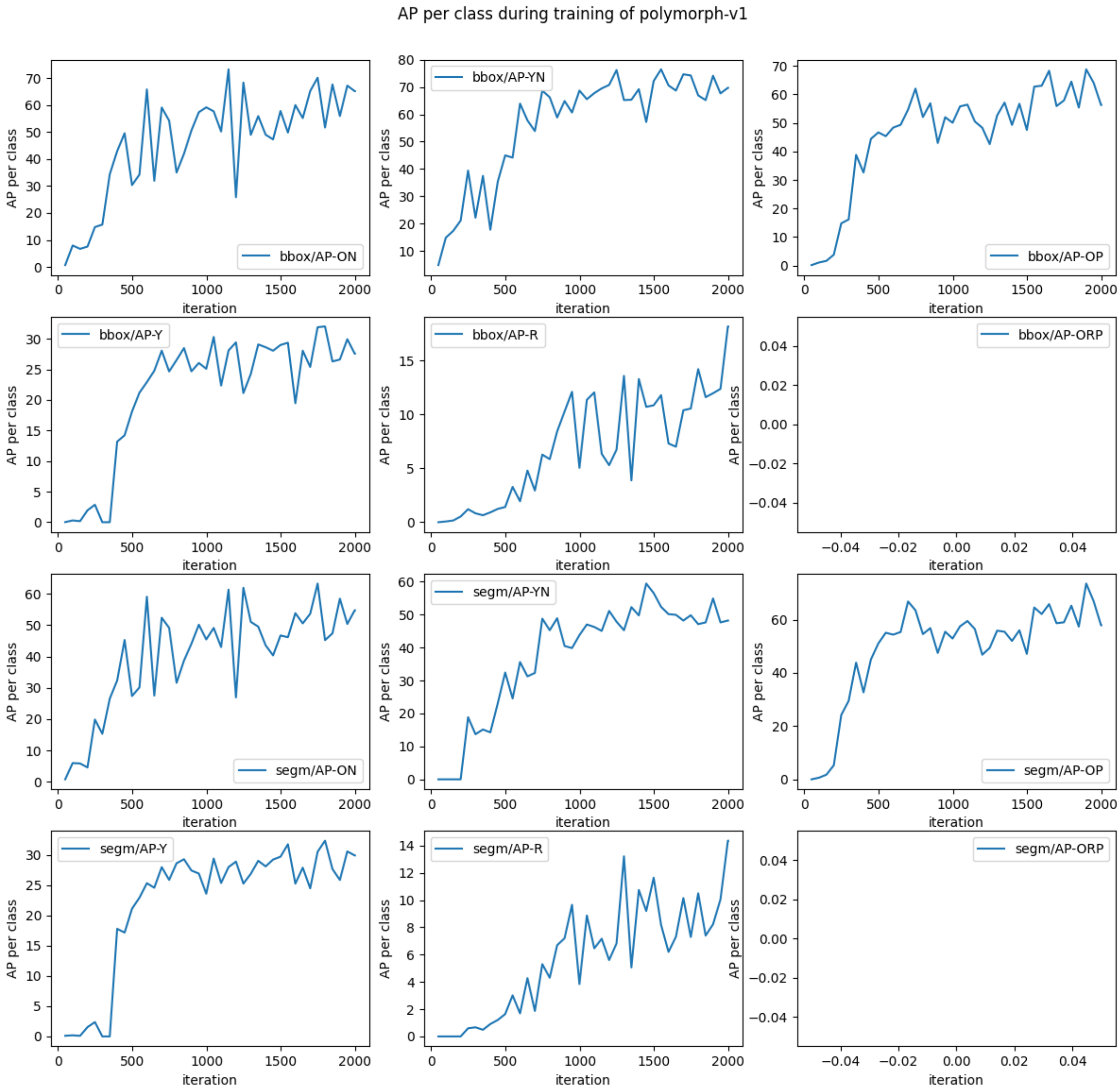

Figure 25. Training metrics for ROY polymorph detection. Average precision values for the bounding boxes and segmentations

3.3 Bayesian Algorithm

Additional results on the implementation of Bayesian Algorithm.

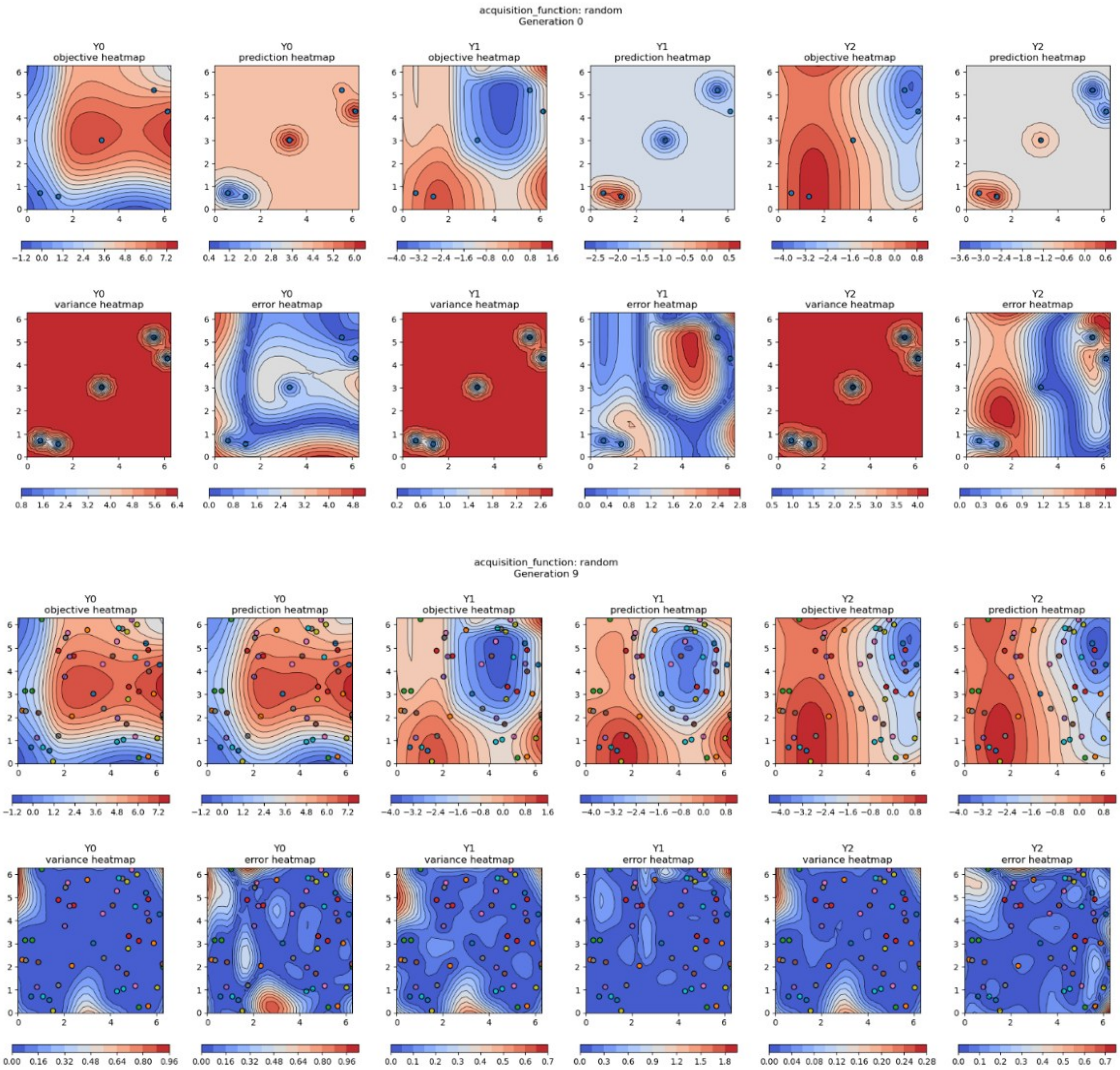

Figure 26. Simulation of the first generation of a random algorithm across three outcomes, each with different ground truth functions: Y0, Y1 and Y2. Top left of each outcome shows the ground truth likelihood of each outcome, and location of samples taken from each space. Top right of each outcome shows the predicted space computed after samples have been taken. Bottom left of each outcome shows the computed uncertainty (variance) of each point in the space. Bottom right shows the difference (error) between the ground truth and predicted outcomes. Upper image shows first generation. Lower Image shows final generation.

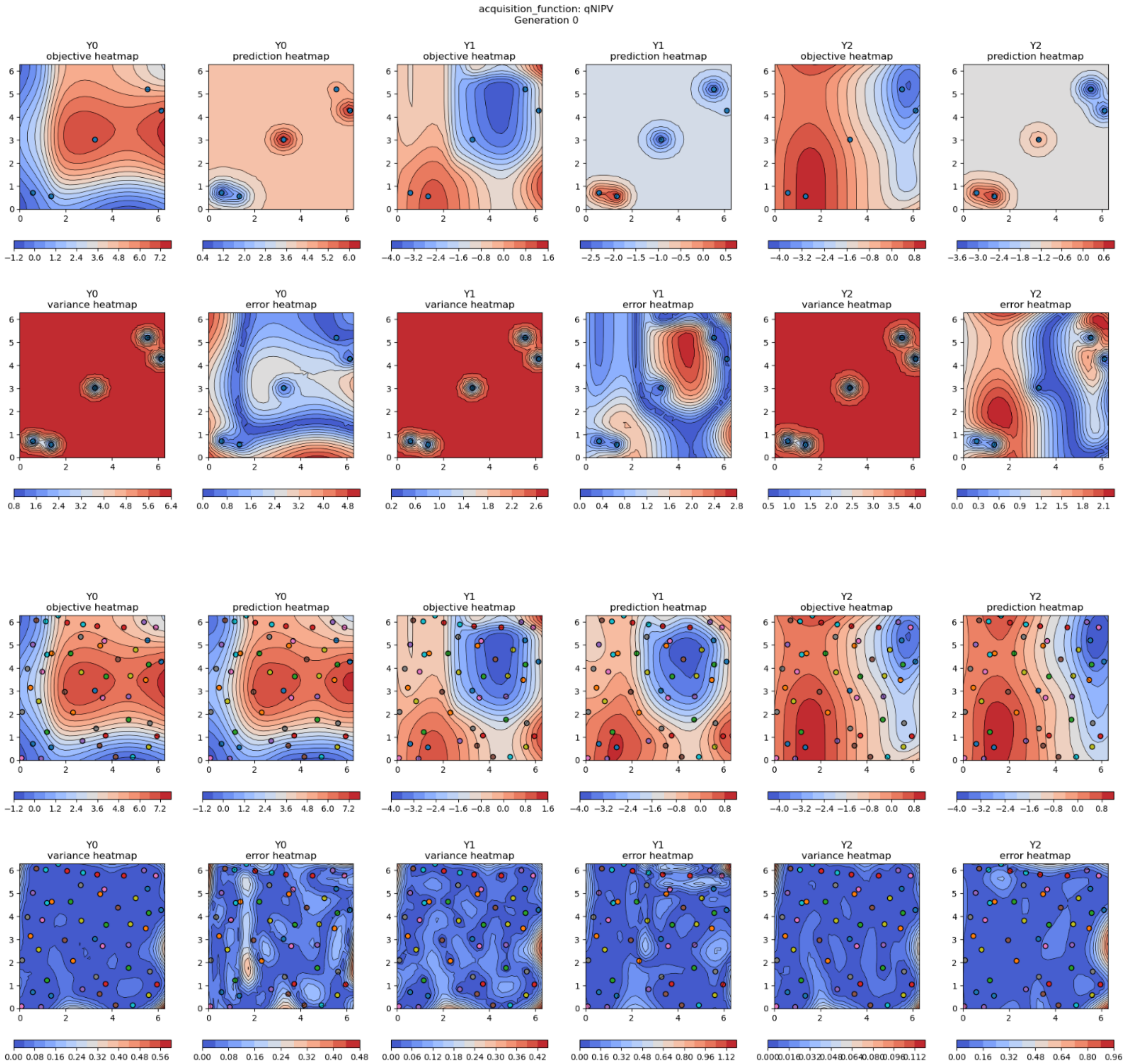

Figure 27. Simulation of the first generation of a Bayesian exploration algorithm across three outcomes, each with different ground truth functions: Y0, Y1 and Y2. Top left of each outcome shows the ground truth likelihood of each outcome, and location of samples taken from each space. Top right of each outcome shows the predicted space computed after samples have been taken. Bottom left of each outcome shows the computed uncertainty (variance) of each point in the space. Bottom right shows the difference (error) between the ground truth and predicted outcomes. Upper image shows first generation. Lower Image shows final generation.

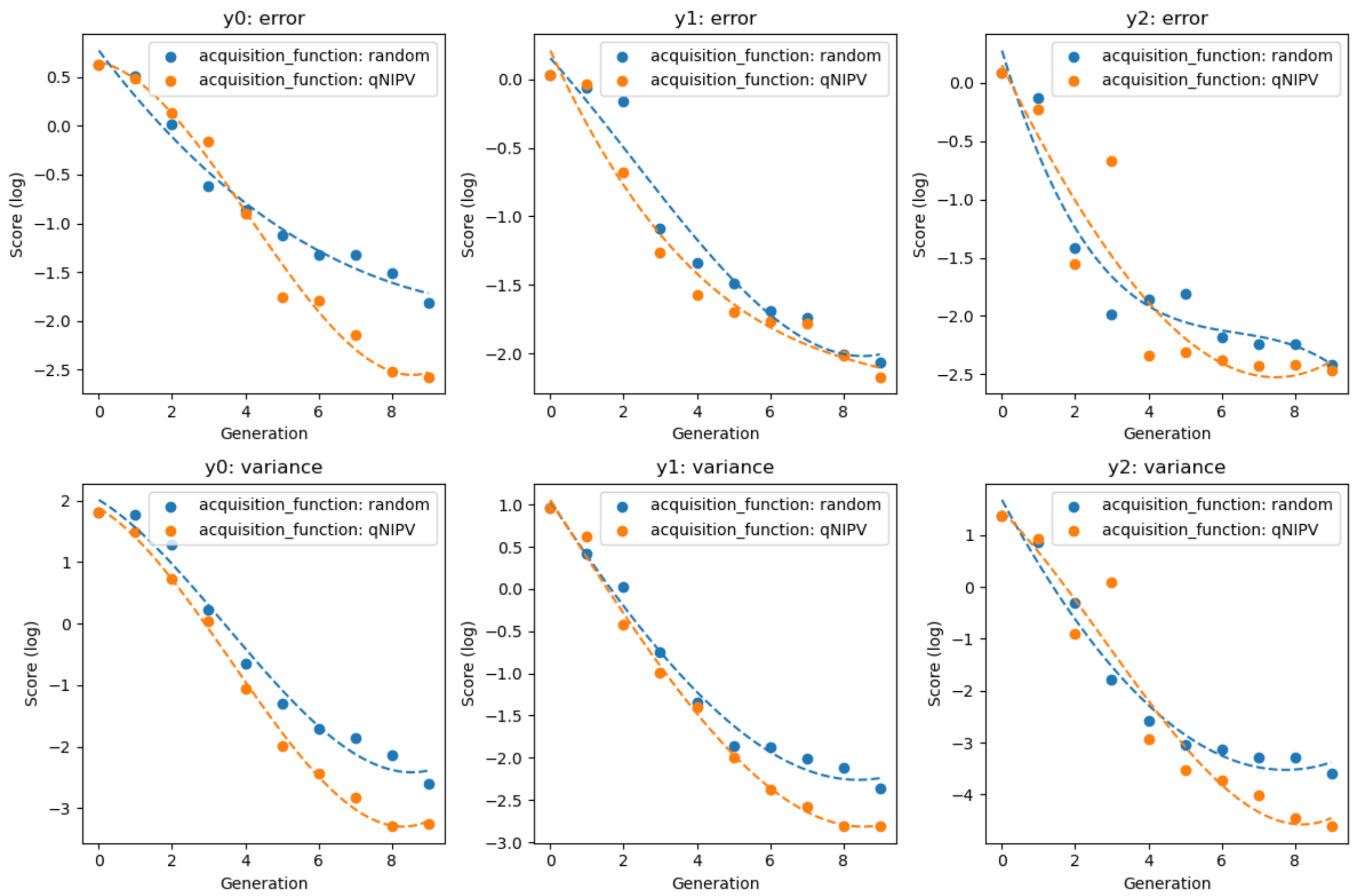

Figure 28. Comparison of model error and variance between generations created by qNIPV and random methods for each simulated polymorph**.** Top row: plot of calculated error in each generation of the simulated experiment for each outcome for each acquisition method. Model error drops lower faster when using qNIPV when compared with random algorithm. Bottom row: plot of calculated variance in the predicted model in each generation of the simulated experiment for each outcome for each acquisition method. The variance is seen to be lower at a faster rate in qNIPV than in random.

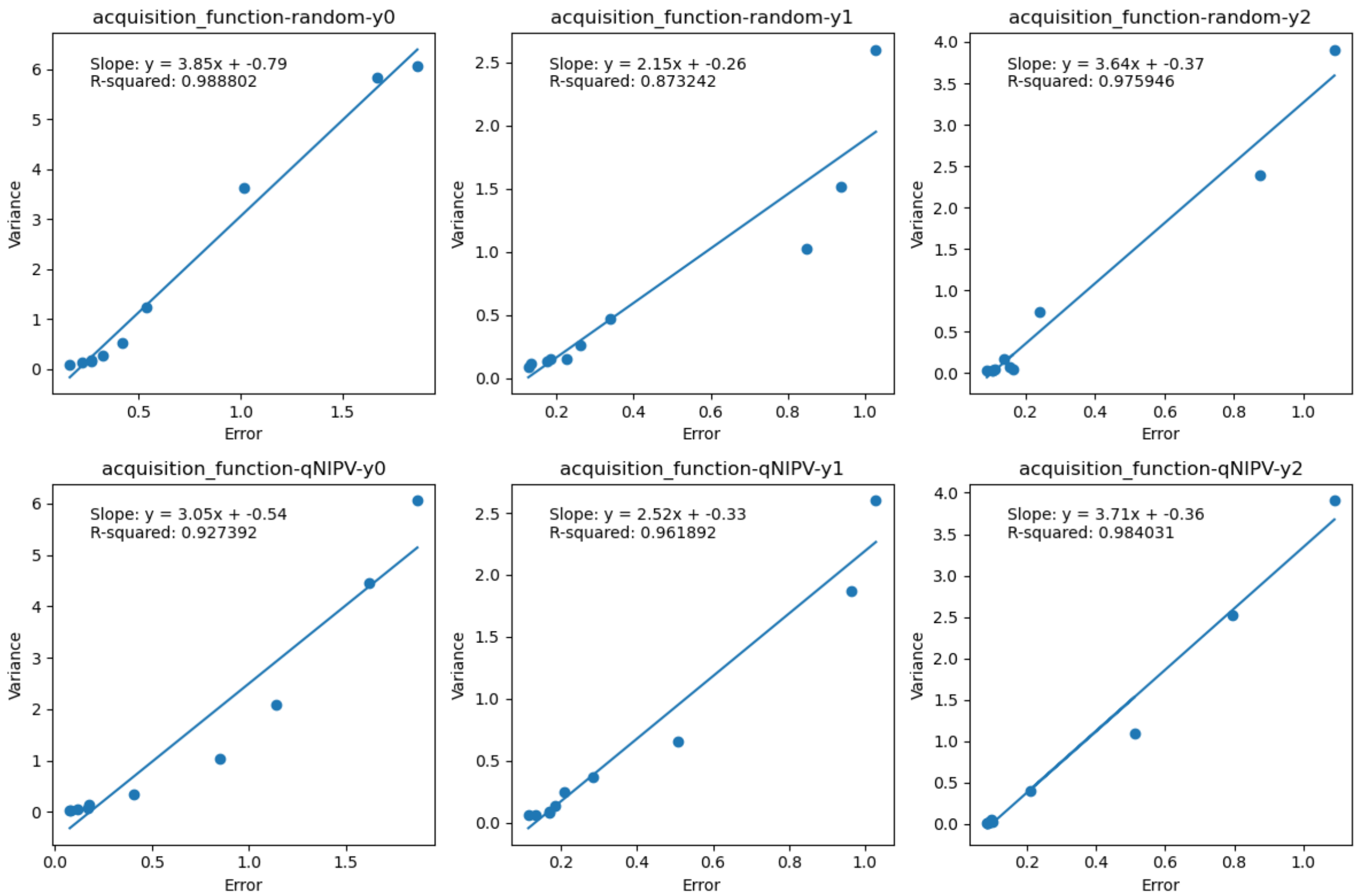

Figure 29. Correlation between error and variance for each polymorph and each acquisition function**.** Each datapoint represented the calculated error and predicted variance in each generation for both acquisition methods. The R-squared value for each plot shows a close correlation between error and variance, suggesting that variance is a suitable approximation for model error

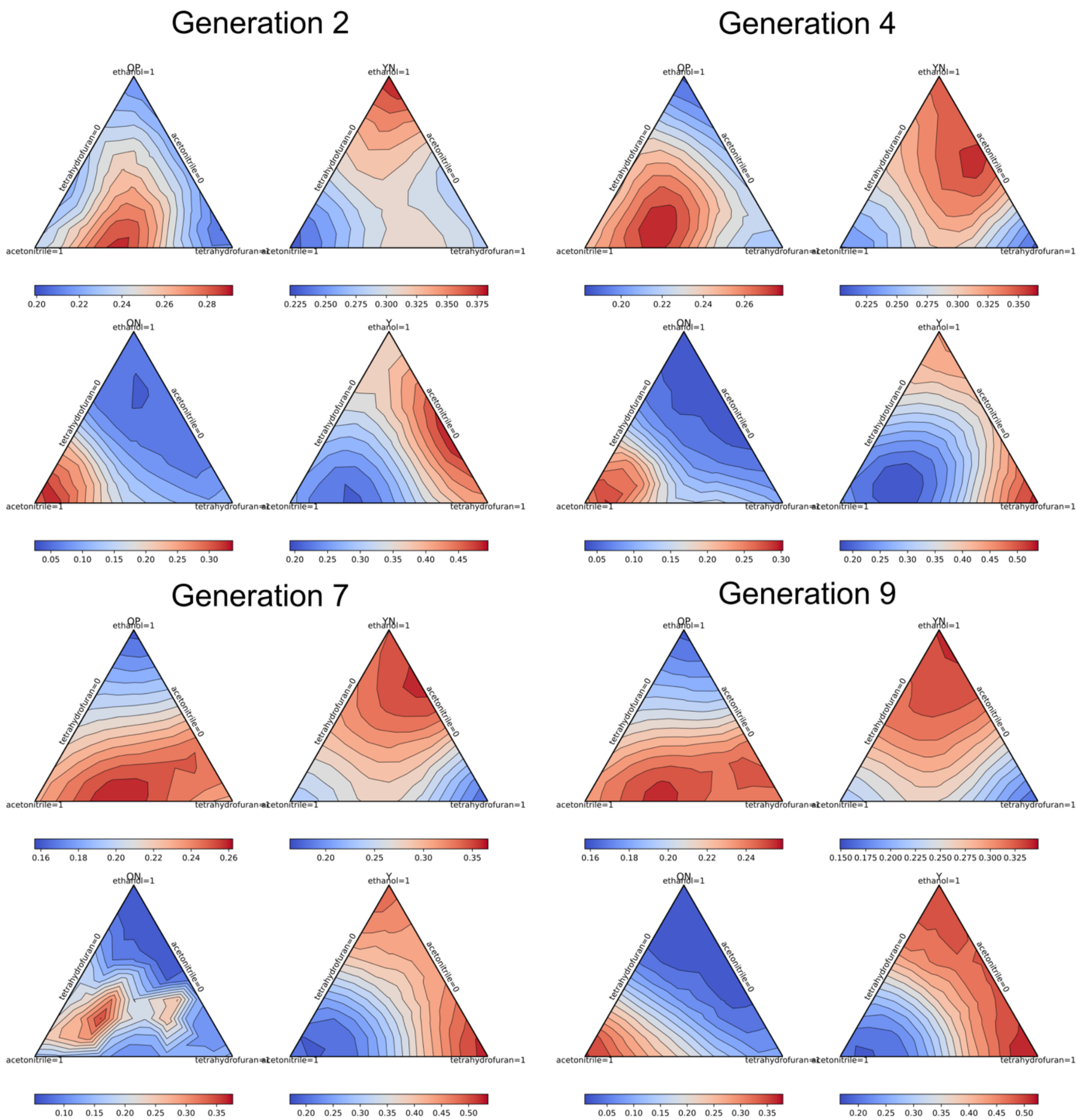

Figure 30. Likelihoods. Simulated likelihood heatmap for each polymorph under different solvent conditions across generations. Four generations are shown here, 2, 4, 7, and 9.

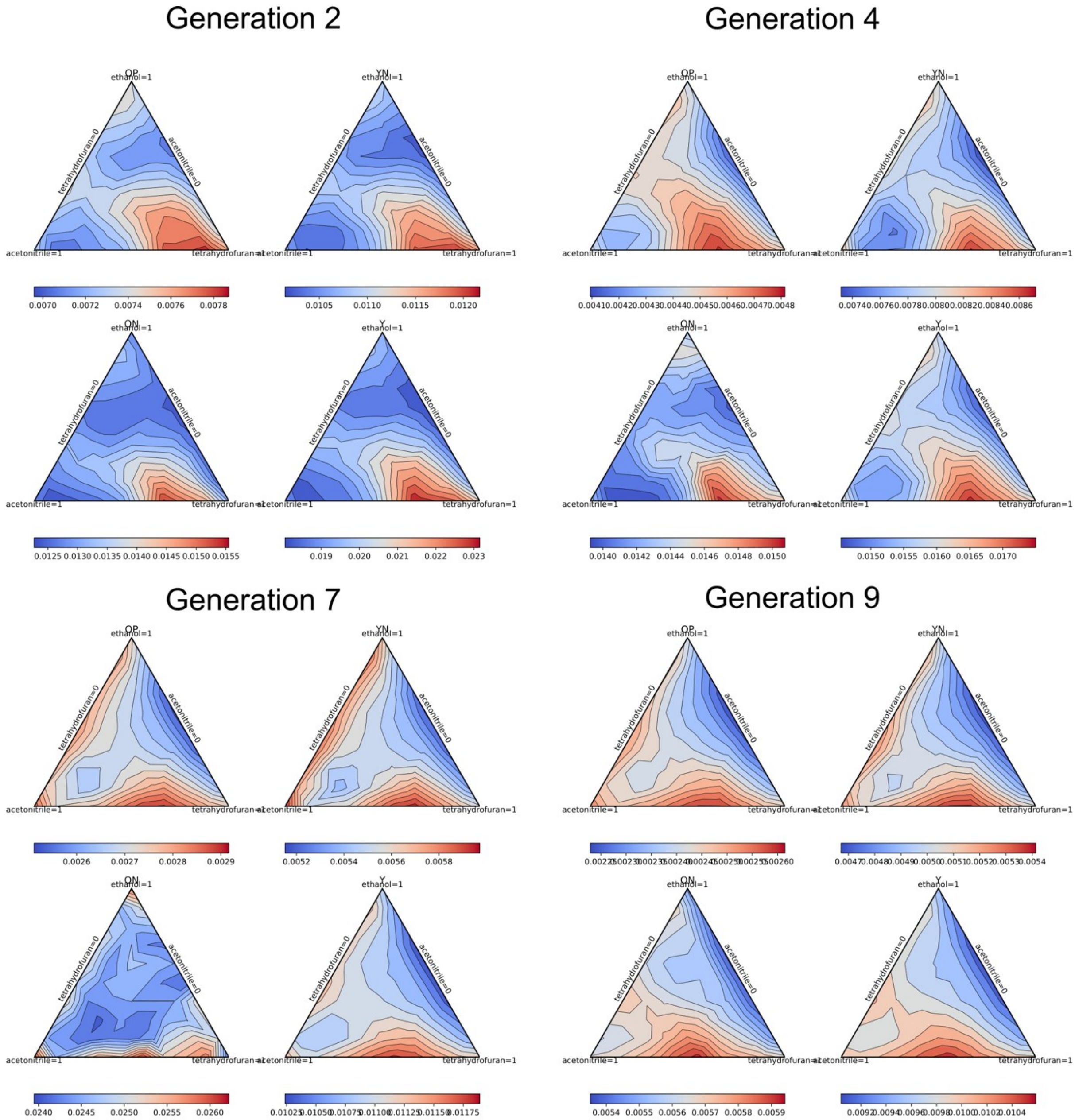

Figure 31. Uncertainties. Simulated uncertainty heatmap for each polymorph under different solvent conditions across generations. Four generations are shown here, 2, 4, 7, and 9.

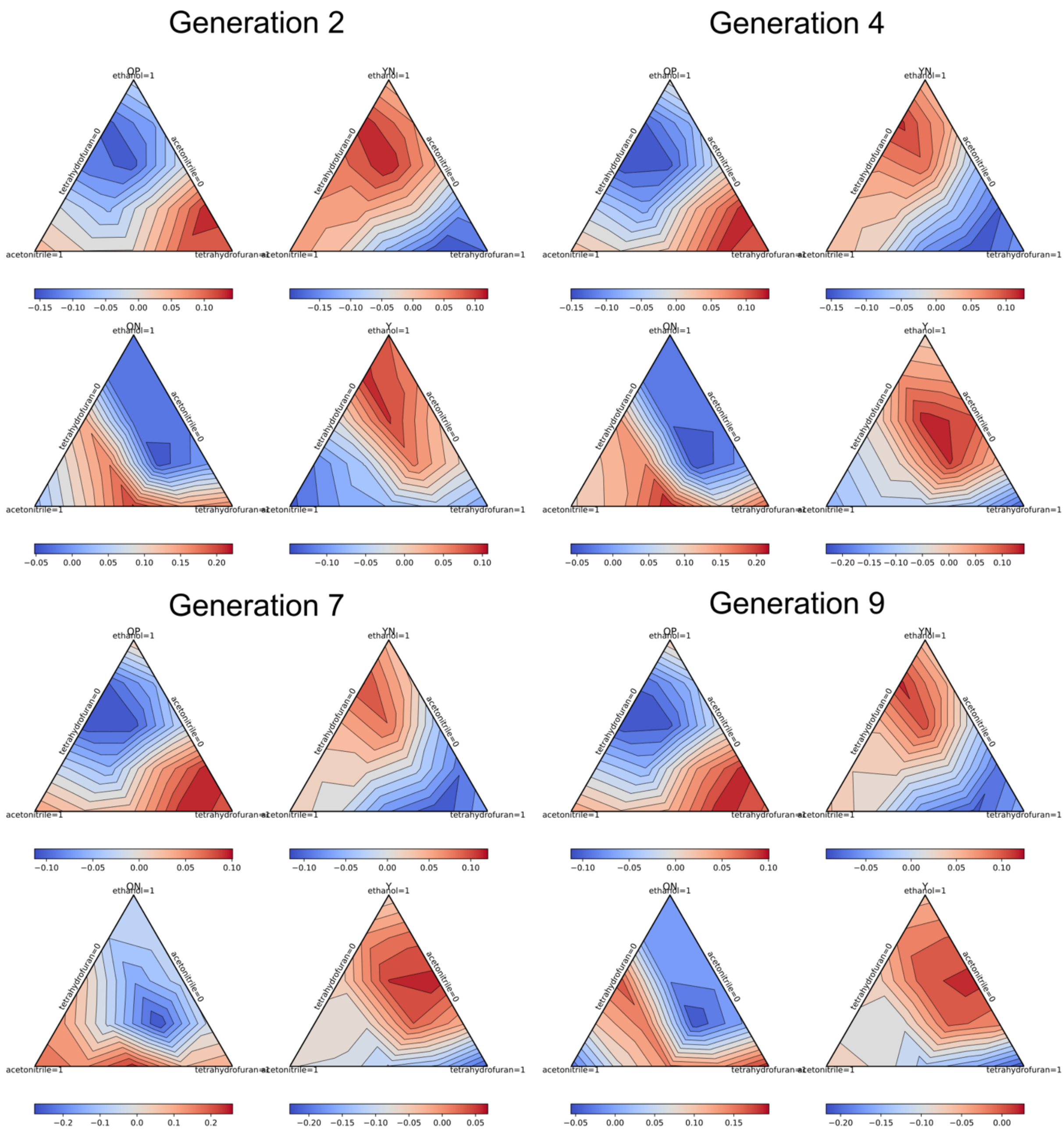

Figure 32. Errors. Simulated error heatmap for each polymorph under different solvent conditions across generations. Four generations are shown here, 2, 4, 7, and 9.

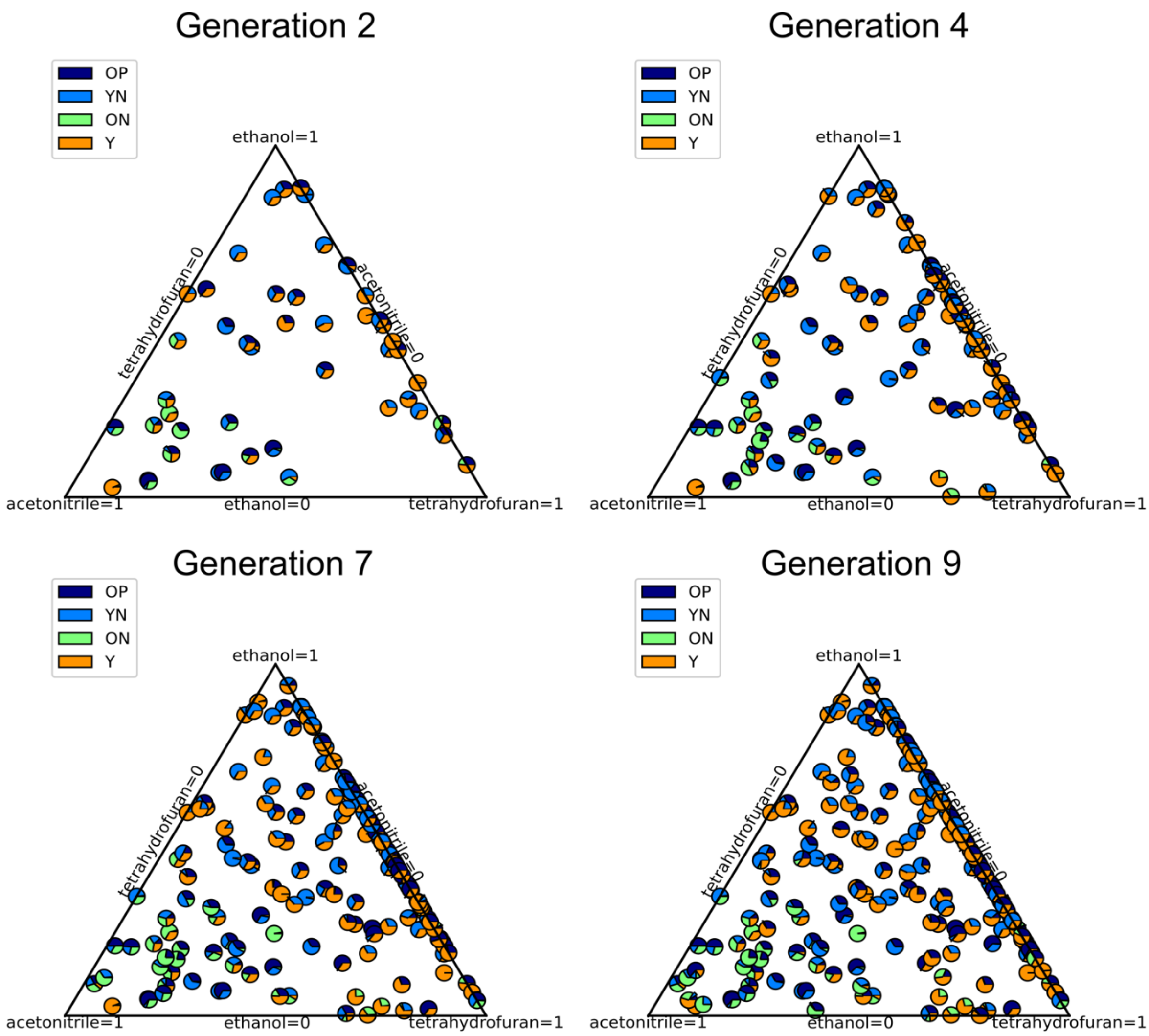

Figure 33. Scores. Simulated scores for each polymorph under different solvent conditions across generations. Four generations are shown here, 2, 4, 7, and 9.

4. References

1. Salley, D. S., Keenan, G. A., Long, D.-L., Bell, N. L. & Cronin, L. A Modular Programmable Inorganic Cluster Discovery Robot for the Discovery and Synthesis of Polyoxometalates. *ACS Cent. Sci.* **6**, 1587–1593 (2020).

2. Jiang, Y. *et al.* An artificial intelligence enabled chemical synthesis robot for exploration and optimization of nanomaterials. *Sci. Adv.* **8**, eabo2626 (2022).
